# Analysis of Diabetes Apps to Assess Privacy-Related Permissions: Systematic Search of Apps

**DOI:** 10.2196/16146

**Published:** 2021-01-13

**Authors:** José Javier Flors-Sidro, Mowafa Househ, Alaa Abd-Alrazaq, Josep Vidal-Alaball, Luis Fernandez-Luque, Carlos Luis Sanchez-Bocanegra

**Affiliations:** 1 Information Systems Department Consorci Hospitalari Provincial de Castelló Castelló de la Plana Spain; 2 Division of Information and Computing Technology College of Science and Engineering Hamad Bin Khalifa University Doha Qatar; 3 Health Promotion in Rural Areas Research Group Gerència Territorial de la Catalunya Central Institut Català de la Salut Sant Fruitós de Bages Spain; 4 Unitat de Suport a la Recerca de la Catalunya Central Fundació Institut Universitari per a la recerca a l'Atenció Primària de Salut Jordi Gol i Gurina Sant Fruitós de Bages Spain; 5 Salumedia Labs Sevilla Spain; 6 Adhera Health Inc Palo Alto, CA United States; 7 Faculty of Health Sciences Universitat Oberta de Catalunya Barcelona Spain

**Keywords:** diabetes mellitus, privacy, mobile apps, dangerous permissions

## Abstract

**Background:**

Mobile health has become a major vehicle of support for people living with diabetes. Accordingly, the availability of mobile apps for diabetes has been steadily increasing. Most of the previous reviews of diabetes apps have focused on the apps’ features and their alignment with clinical guidelines. However, there is a lack of knowledge on the actual compliance of diabetes apps with privacy and data security guidelines.

**Objective:**

The aim of this study was to assess the levels of privacy of mobile apps for diabetes to contribute to the raising of awareness of privacy issues for app users, developers, and governmental data protection regulators.

**Methods:**

We developed a semiautomatic app search module capable of retrieving Android apps’ privacy-related information, particularly the dangerous permissions required by apps, with the aim of analyzing privacy aspects related to diabetes apps. Following the research selection criteria, the original 882 apps were narrowed down to 497 apps that were included in the analysis.

**Results:**

Approximately 60% of the analyzed diabetes apps requested potentially dangerous permissions, which pose a significant risk to users’ data privacy. In addition, 28.4% (141/497) of the apps did not provide a website for their privacy policy. Moreover, it was found that 40.0% (199/497) of the apps contained advertising, and some apps that claimed not to contain advertisements actually did. Ninety-five percent of the apps were free, and those belonging to the “medical” and “health and fitness” categories were the most popular. However, app users do not always realize that the free apps’ business model is largely based on advertising and, consequently, on sharing or selling their private data, either directly or indirectly, to unknown third parties.

**Conclusions:**

The aforementioned findings confirm the necessity of educating patients and health care providers and raising their awareness regarding the privacy aspects of diabetes apps. Therefore, this research recommends properly and comprehensively training users, ensuring that governments and regulatory bodies enforce strict data protection laws, devising much tougher security policies and protocols in Android and in the Google Play Store, and implicating and supervising all stakeholders in the apps’ development process.

## Introduction

### Background

Diabetes mellitus (DM) is one of the most common chronic conditions around the globe. The number of people with DM has risen globally from 108 million in 1980 to 422 million in 2014 [1]. Its prevalence has been increasing everywhere, especially in middle-income countries, from 4.7% in 1980 to 8.5% in 2014. DM increases the risk of serious health problems such as myocardial infarction, renal failure, stroke, and lower limb amputation [2]. Diabetic retinopathy is one of the most important causes of blindness worldwide, especially in developed countries [3]. DM has also been linked to an increased risk of other conditions such as dementia, depression, and some types of cancer [4]. In order to reduce the risk of complications, intensive patient education and support are needed, which can be enhanced by the use of mobile technology.

Along with the exponential increase in the number of health apps [5,6], in particular the number of diabetes apps has increased significantly in the last several years [7]. Mobile health (mHealth) has become a major vehicle of support for people living with diabetes, and the availability of mobile apps for diabetes has been steadily increasing. Most of the previous reviews of diabetes apps have focused on their features and their alignment with clinical guidelines [8,9]. However, there is a lack of knowledge on the actual compliance of diabetes apps with privacy and data security guidelines.

Therefore, there is a growing concern to review diabetes apps because in many cases they do not possess the quality and content that they should according to their own declared purposes [10,11]. In addition, some studies that have investigated the effectiveness of mobile apps clearly demonstrate data privacy problems [12], as well as a lack of transparency with the provided information [13].

Studies on mHealth and privacy have raised some serious concerns in recent years. Because very sensitive information is increasingly accessed and shared using mobile apps, there is an obvious need for clinicians, software developers, users, and patients to be aware of and trained on information privacy aspects. Personal data may be collected through different means, such as being entered directly by the user or being recorded by the phone’s camera, microphone, or paired wireless device (eg, Bluetooth glucometer apps). It is crucial to note that the treatment of these critical data demands a special approach regarding security and privacy. However, some apps do not even provide information regarding their privacy policies. In some instances, these privacy terms are difficult to understand by nontechnical users, and some privacy policies may even be regarded as abusive. To make matters worse, the ecosystem of mobile apps is so complex that even app developers and users may not know with whom the data is being shared and for what purpose [14-16].

An additional challenge is that very often stakeholders are not involved in the app development process and consequently cannot provide feedback on privacy preferences [10].

To deal with these issues, some researchers such as Stoyanov et al [17] have attempted to develop a suitable framework—the Mobile App Rating Scale—that allows for the evaluation of the quality of apps. Alternatively, other investigations have focused specifically on privacy or legal issues [18]. In the case of mHealth for diabetes, recent reviews looked into aspects linked to the efficacy of interventions [19,20] but did not address aspects related to privacy. Other research has investigated privacy aspects in generic mHealth apps [12,21]. However, to the best of our knowledge, this study is the first to focus on investigating privacy issues and dangerous permissions in diabetes mobile apps. Studies looking at diabetes apps have not conducted in-depth analyses of dangerous permissions on the Android platform [22].

### Objectives

The aim of this study was to evaluate the privacy-related permissions of Android diabetes apps in Google's Play Store using a semiautomatic approach that relies on the extraction of privacy-related features (eg, permissions, terms of usage). This approach was designed to assist in identifying strategies to raise the awareness of app users, patients, and clinicians. To illustrate our approach, we provide two case studies of diabetes apps that were comprehensively analyzed (Multimedia Appendix 1).

## Methods

### Study Design

The first step in this study was the extraction of metadata from mobile apps’ metadata using a web-based application programming interface (API) [23]. We used the platform 42Matters, which offers a web-based commercial tool that facilitates access to the Android Google Play Store and to other mobile platforms’ apps’ metadata through a proprietary API [24]. Searches were conducted with the developed script module 42Matters’ index of Android apps. Since the 42Matters platform did not allow the extraction of privacy-related permissions from Apple’s App Store, the research centered on Android apps from Google’s Play Store. Data extraction was focused on potentially dangerous permissions [25] that allow the requesting app access to private user data or control over the mobile device, both of which can negatively impact the user. Because this type of permission introduces potential risk, the system does not automatically grant it to the requesting app. Our methodology was based on similar studies of health apps that used the 42Matters platform, but focusing on privacy-related information [26,27].

In order to complement the quantitative results already presented, we described and investigated two very popular and well-rated diabetes apps (presented in Multimedia Appendix 1) from a qualitative perspective.

For the extraction of the diabetes apps’ metadata, we first devised the architecture [28] and subsequently developed the corresponding software module for the automatic extraction of mobile app metadata using the web-based API of 42Matters. The output of this module is a data set stored locally in a comma-separated values (CSV) file. The source code for the module was released under the GNU AGPLv3 license and can be found on the GitHub link [29]. This module is capable of querying the API of the 42Matters platform to retrieve metadata related to diabetes apps, including the Android permissions required by the apps. The module was designed to extract apps with the following search parameters: (1) language (we searched for English-language apps), (2) keyword search (we searched for apps whose titles included the root words “diabet” and “mellitus”), and (3) app categories (we selected the categories medical, health and fitness, lifestyle, and education).

The resulting apps were manually reviewed (see Multimedia Appendix 1) to assess whether they were related to diabetes. All apps were related to diabetes, but we did not address the quality of their content. As explained in the “Limitations” section, choosing a method where search fields matched the description—and not only the title—would have resulted in more apps, many of which would not have been related to diabetes.

**Figure 1:**
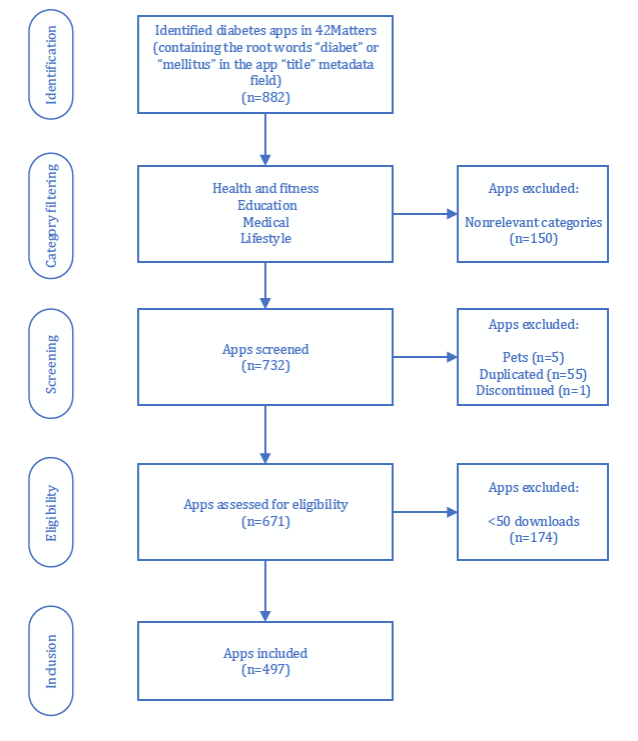
App selection process flowchart.

Once the most suitable app categories were identified, it was then possible to move on to design the entire app selection process, which consisted of the following steps (see Figure 1):

- Step 1: “Identification” phase—all of the diabetes apps that contained the root words “diabet” or “mellitus” in an app’s title field were selected, resulting in 882 apps; by matching diabet or mellitus, it was possible to ensure that any relevant potential variations of the words that contained these root words (ie, diabetes, diabetic, diabetics, mellitus, etc) were included in the search.
- Step 2: “Category filtering” phase—in order to guarantee that only relevant diabetes apps were included in the study, all the retrieved apps that did not belong to the medical, health and fitness, education, or lifestyle categories [30] were automatically filtered out by the 42Matters script module and excluded from the study; this filtering resulted in 732 apps.
- Step 3: “Screening” phase—in this phase, we manually filtered apps and excluded 5 diabetes apps related to pets, 1 discontinued app, and 55 duplicated apps; this screening resulted in 671 apps.
- Step 4: “Eligibility” phase—we excluded apps that did not have a minimum of 50 downloads, and therefore discarded 174 apps.
- Step 5: “Inclusion” phase—the resulting 497 apps were analyzed, which were the objects of analysis of this research.

### Data Extraction: Retrieved Metadata Fields

After the final set of apps was selected in June 2019, a process was initiated to extract all the relevant metadata and information, which were stored in a CSV file. All the retrieved fields are described in the table below.

**Table 1:** Description of apps’ retrieved metadata as provided by 42Matters.

App’s metadata field	Description
Title	Main name of the app
Price	Price and currency (0 if it was free)
Permission	Required Android permissions of the app
Rating	App’s average rating from 0 to 5 (0=worst, 5=best)
Number of downloads	Number of times the app was downloaded
Number of ratings	Number of times the app was rated
Contains advertising	True if the app contained advertising and false if it did not
Category	Category to which the app belonged (medical, health and fitness, education, or lifestyle)
Short description	Short description of the app’s declared purpose
Website	Website of the app
Privacy policy	Website showing the app’s privacy policy

### Extraction of Android Privacy-Related Permissions

Starting with Android 6.0 (API 23 level), users grant permissions to apps while using them, not when an app is installed. On the one hand, this approach simplifies the process of installing the app because the user does not need to grant permissions when installing or updating the app. In addition, it provides the user with more control over the app’s functionalities because users can revoke the granted permissions from the app’s configuration screen at any time. On the other hand, this new approach complicates the app’s usability because dangerous permissions have to be granted while using the app, which poses an additional challenge for untrained users. Android distinguishes between 4 categories of permissions: normal, signature, dangerous, and special [31].

Signature and special permissions will not be explained here because they are rarely used and were not found in any of the apps included in our research. The most frequently requested permissions are normal and dangerous permissions. If an app declares a normal permission in its manifest, the system grants permission to it automatically without the user’s intervention. On the other hand, Android considers dangerous permissions as critical because they allow apps to access users’ critical data. More concretely, an Android dangerous permission [25,32] allows the requesting app access to private user data or control over the mobile device. Because this type of permission allows developers to access users’ data, photos, and videos stored on the device, it introduces potential risk, and the system does not automatically grant it to the requesting app [33,34].

In brief, normal permissions do not put the user’s privacy at risk directly. Consequently, if an app declares a normal permission in its metadata, the system grants permission to it automatically without the user’s intervention. On the other hand, a dangerous permission allows an app to access the user’s critical data, and consequently the user should explicitly authorize this permission [35]. The 10 most required dangerous permissions found in this research are shown in Multimedia Appendix 2.

## Results

### App Functions

The process described in the “Methods” section retrieved a total of 497 apps (Multimedia Appendix 3). The breakdown of privacy-related permissions is summarized in Table 2. Most of the apps required at least one dangerous permission.

**Table 2:** Summary of the privacy-related main features of retrieved diabetes apps.

Assessed parameter	Diabetes apps (N=497), n (%)
Does not require any permissions (either normal or dangerous)	89 (17.9)
Only requires normal permissions	111 (22.3)
Requires at least one dangerous permission	297 (59.8)
Does not provide a website link to its privacy policy	141 (28.4)
Contains advertising	199 (40.0)

The reason for apps not requesting any permissions is that they serve very basic functions (eg, calculators, logs, diaries, etc) that only need access to very basic and noncritical Android resources. Only 22.3% (111/497) of the apps required normal (noncritical) permissions alone. On the other hand, 59.8% (297/497) of the apps required at least one dangerous permission. This might be partially justified by these apps’ more advanced functionalities (eg, doctor-patient interaction, connecting to a glucometer, calorie-burning calculation, scanning the barcode of diabetic food, etc).

Regarding privacy, it was worrying to discover that 28.4% (141/497) of the apps did not return the privacy policy metadata field, consequently posing additional difficulty for users to adequately understand how these apps would treat very sensitive personal information.

Finally, 40.0% (199/497) of the apps contained advertising, which can imply the sharing of critical personal data (eg, a user’s precise location) with unknown third parties for geolocated advertisement. Consequently, because the advertising business model in the mobile ecosystem is usually linked to the sharing or selling of critical personal data [36], the aforementioned findings unquestionably confirm the necessity to educate users and raise awareness regarding user privacy in diabetes apps.

### Dangerous Permissions

As explained below, dangerous permissions refer to permissions that might lead to data breaches of private information [37]. From the 497 diabetes apps included in our final analysis, a substantial number of them—297 (59.8%)—required dangerous permissions. Table 3 shows, in decreasing order, which dangerous permissions were most frequently requested by the apps.

**Table 3:** Summary results of apps with the requested privacy-related permissions.

Dangerous permission	Diabetes apps that requested it (N=497), n (%)
Write external storage	272 (54.7)
Read external storage	169 (34.0)
Access coarse location	103 (20.7)
Access fine location	95 (19.1)
Camera	89 (17.9)
Get accounts	82 (16.5)
Read phone state	81 (16.3)
Record audio	39 (7.8)
Call phone	23 (4.6)
Read contacts	22 (4.4)
Others (the sum of the remaining dangerous permissions)	28 (5.6)

**Figure 2:**
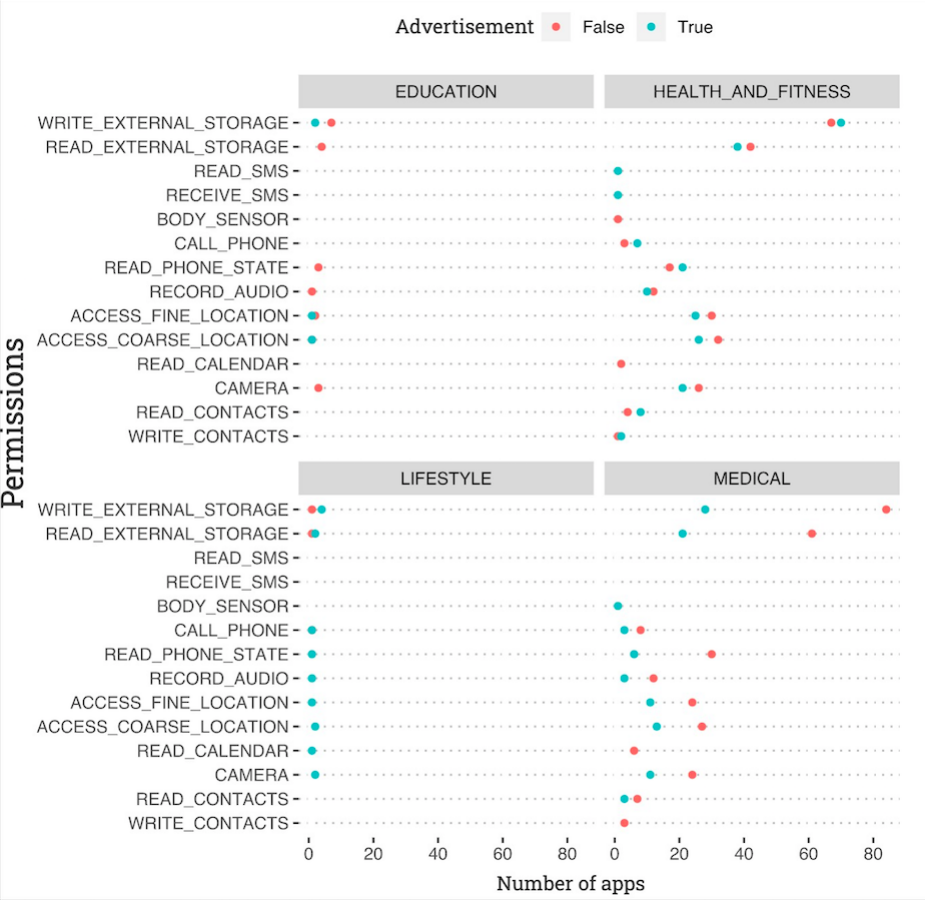
The top 14 dangerous permissions by app category (lifestyle, medical, education, and health and fitness) and type of privacy-related permission requested, as well as whether they included advertising (“True”) or not (“False”).

In addition, Figure 2 illustrates the number of apps that required each of the top 14 dangerous permissions, arranged by category. The four quadrants represent each of the four categories to which the apps belonged: education, health and fitness, medical, and lifestyle. In addition, the “Advertising” tag indicates whether an app contained advertising: the ones in blue contained advertising, while the ones in red did not. The x-axis shows the number of apps, while the y-axis lists the 14 most requested dangerous permissions.

## Discussion

### Principal Results and Comparison With Previous Work

Although we identified the apps requesting access to the camera (89/497, 17.9%), we need to study the actual usage of apps in order to fully understand the context before we consider that access to be a potential risk. For instance, in the case of diabetes, it is very common to use the camera for food logging. On the other hand, except for advertising or fitness tracking (eg, calorie counting), the need for the user’s geolocation data seems difficult to justify. In this sense, what might be acceptable in one app might not be reasonable in others. Similar studies found that 77 of 186 (41.4%) permissions requested by 58 popular German mHealth apps were not related in any way to the apps’ functionalities [38]. Moreover, 15 of 42 (35.7%) Android health and well-being apps accredited by the UK’s NHS Health Apps Library requested critical permissions for unjustifiable reasons [12]. Similarly, other research concluded that several popular mental health apps and mHealth apps requested permissions that were not aligned with the apps’ stated purposes [14,21]. One of the consequences of requesting unnecessary dangerous permissions is a decrease in users’ trust, acceptance, and use of these apps.

Another finding of this study was that 95.4% of the apps were free of charge. The business model of free apps is, in most cases, based on advertising (through services such as Google AdMob), resulting in the disclosure of users’ critical data, either directly (through the app itself) or indirectly (through Google’s commercial advertising platforms).

The reliance on advertising of some of the studied apps might be linked to the high number of apps requesting geolocation, since location can increase advertisement revenue. A study on NHS-accredited apps found some evidence that patients’ data were information for advertisers [12]. Other studies also found that users’ information was shared in 19 of 24 popular medication-related apps in the United Kingdom, the United States, Canada, and Australia [39]. Research of privacy in the top 36 mental health and smoking cessation apps also found a lack of compliance with disclosing or sending data to third-party providers [40]. Although app developers usually claim that they do not collect or share personally identifiable data, users can be easily identified by correlating advertising services using data analytics [39].

In addition, 28.4% of the studied apps did not provide a privacy policy website, which corroborates results from other research that demonstrated that 48% of 17,991 free Android apps did not have a privacy policy [18]. Building on this finding, 81% of 154 Android apps related to hypertension and diabetes did not refer to a privacy policy [33]. In addition, a privacy policy was missing in 417 of 600 (69.5%) prominent mHealth apps [41]. Most likely, had we not discarded less reliable apps in our research, the percentage of apps that did not provide a link to a website with their privacy policy would have been higher [34]. The lack of a privacy policy is a critical fault, as it prevents users from properly understanding how apps treat their very sensitive personal information. Further, the discrepancy between apps’ privacy policies and their actual features has been reported in several studies [12,18]. This issue might be partially attributed to the fact that app developers have insufficient knowledge about privacy best practices [42].

In our study, 59.8% of apps required at least one dangerous permission, the two most requested being write external storage (54.7%) and read external storage (34.0%). This finding confirms the results from previous research. For instance, the most common dangerous permissions requested by the most popular freeware mHealth apps were write external storage (90%) and read external storage (50%) [34]. For prominent mental health apps in the Google Play Store, the most frequently requested permissions were also write (73%) and read (73%) external storage. In addition, these two permissions were the most requested (79%) in medicine-related apps in the Google Play Store in the United Kingdom, the United States, Canada, and Australia [38]. These permissions may indeed jeopardize users’ privacy because they allow developers to access users’ data, photos, and videos stored on the device [33,34]. Another relevant finding was that health and fitness apps usually requested more dangerous permissions than apps belonging to other categories [21].

Apps’ ever-changing functionality and privacy policies, as well as their complexity, do not facilitate matters, either. Moreover, having to manually accept dangerous permissions when using an app poses an additional challenge that can have detrimental consequences, particularly for less knowledgeable users. For instance, individuals with low literacy rates or the elderly would require adequate training to truly understand what they are consenting to before using diabetes apps. Existing tools to evaluate eHealth literacy skills [43] do include security awareness as one of their dimensions. However, the complexity of potential security issues is increasing, and it might be necessary to develop new tools and training methods for both patients and health care providers.

### Practical Implications

These findings have very important practical implications for users, physicians, developers, and policy makers [44,45]. To select an appropriate mobile app for diabetes, end users should be aware of what type of personal data is collected, used, and shared by a certain app by carefully reading the app’s description, terms of use, and privacy policy.

In addition, it is imperative to emphasize the need for training so that users are able to understand complex privacy policies and terms of service and are fully aware of the privacy risks derived from the sharing of their data with third parties. Users should also be knowledgeable about the different types of dangerous permissions so that they can discern how each particular permission may jeopardize their data. The ultimate goal is to empower users so that they can autonomously and proficiently deny access to any unjustifiable dangerous permission.

To minimize the privacy risks derived from using diabetes apps, savvy users should use AdBlock or encryption apps [33]. Moreover, health care providers should ensure that the apps they recommend to patients adhere to a strict privacy code, and they should assist users in selecting suitable apps by explaining both the apps’ benefits and their risks.

App developers should enforce their apps’ full compliance with internationally recommended standards and practices [46-49]. Specifically, developers must ensure that their apps’ privacy policies are always readily available, very simple to read, and able to be understood by any user. Further, their apps should never request dangerous permissions not directly related to the apps’ declared purpose. Developers should not—without the users’ explicit consent—collect, use, or share user data for any purpose outside of the predefined scope of the app, and all data sharing practices should be transparently disclosed to users. Last but not least, developers should be aware of diverse privacy laws and data protection legislation, which differ greatly depending on the country or region of use.

In terms of privacy laws, apps tend to adhere to the data protection legislation in the developers’ country of origin but not in the apps’ country of use. Therefore, regulators around the world should collaborate to establish a specific international accreditation program for diabetes apps. Such a program should be based on unified privacy best practices in which user privacy is the main priority. Because app developers reserve the right to change their privacy policies at any given time and modify their apps’ declared purpose and functionalities, regulators should regularly monitor developers’ adherence to the recommended privacy practices. As well, regulators should emphasize developers’ responsibility and accountability for protecting user data. In addition, app stores should mandate stringent principles and standards that actually compel developers to provide simple and intelligible privacy policies in their apps, especially taking into consideration untrained or illiterate users.

### Limitations

We opted to use the free version of the commercial platform 42Matters instead of the Google Play Store because the Google Play Store had a limit of 250 apps per query.

Another limitation was that the developed module exclusively searched for all diabetes apps that contained the root words diabet or mellitus in the title field. There are some diabetes apps in which the aforementioned root words appear in the app’s description but not in the app’s name. Therefore, some diabetes-related apps may have been excluded from the study. However, this criterion was selected for two principal reasons: (1) to ensure that only truly diabetes-related apps were retrieved, and (2) to make the best use of limited resources (there was neither enough time nor enough labor to thoroughly screen 4700+ apps, many of which bore no relation whatsoever to diabetes). In this sense, our research was not intended to be exhaustive. Rather, we wanted to quantify and evaluate the overall privacy characteristics of the most representative sample of diabetes-related apps. A broader search (ie, to query for all apps that contained the root words diabet or mellitus in the apps’ descriptions) would certainly have yielded many false positives of apps unrelated to diabetes and hence required a very resource-intensive manual screening of the apps, which would have been an unnecessary complication of the overall analysis process.

The study did not comprehensively address either the fact that the number of permissions an app requests does not necessarily reflect how risky the app may be. For instance, an app requesting, unnecessarily, a single dangerous permission, could seriously endanger users’ personal data by collecting and illegitimately sharing them. On the other hand, an app requesting multiple dangerous permissions, but for valid technical or functional needs, could be considered safe. Therefore, the amount of personal information that users are putting at risk depends on many factors, such as the app’s functionality, the permissions it requests, and the context in which these permissions are being used [50]. To perform a more complete assessment of apps’ privacy risks, additional technical, human, and contextual research (eg, analysis of the skills of patients using diabetes apps) should be conducted. For example, when dealing with privacy issues in health apps, an important factor to be considered would be the legitimacy of the request, as highlighted in a recent publication on mHealth apps for cancer in which the authors evaluated a new scale to assess the privacy policies of mHealth apps [51]. Tracking users’ location might be fair in the case of reporting a medical emergency (eg, hypoglycemic crisis).

Although the methodology employed in this research was robust and Google is continuously improving Android and the Play Store’s security policy, this study found evidence that it is extremely difficult to prove whether diabetes apps actually comply with their privacy policies. In fact, even Google cannot control the many malicious apps that are frequently uploaded by hackers in its Play Store and is consequently forced to periodically remove massive numbers of these fraudulent apps [52-54]. Further, a recently published two-year study discovered 2040 potential counterfeit apps that contained malware in the Google Play Store [55].

This study did not cover all of the elements related to the privacy and security of diabetes apps. Privacy protection cannot be guaranteed solely by controlling permissions; for instance, unsecure internet connections can also jeopardize the privacy of mobile app users. Finally, our study only evaluated the apps on one app store; the privacy policies and the requested dangerous permissions in other app stores, such as Apple’s App Store or Samsung's Galaxy Store, might have yielded different outcomes. However, Android’s Google Play Store was also chosen due to its popularity.

### Future Research

A possible expansion of the research could include investigating those diabetes apps that were excluded from this research, either because they belonged to nonrelevant categories or because the developed module did not search for the root words in the apps’ description field. Future research could also focus on analyzing the taxonomy of app categories and match them to officially recognized and standardized clinical categories, such as the Systematized Nomenclature of Medicine Clinical Terms or Medical Subject Headings. Related to that, there is a new trend emerging toward the creation of machine learning approaches to identify privacy issues in mobile apps [56,57]. However, to the best of our knowledge, those methods have unfortunately not yet been applied to health apps. Further, there is a need for homogenous approaches for the assessment of privacy in health apps, as was highlighted recently in a scoping review addressing the issue [58].

Finally, from a legal perspective, although many diabetes apps are available worldwide, their privacy policies usually only comply with the specific national data protection regulations of the developers’ country or region of origin. For instance, the BeatO SMART Diabetes Management app claims that both its privacy policy and its terms of use fully adhere to Indian law, but if this app were to be used in the Middle East or the European Union, it would be unclear whether it would also comply with data protection laws in the country or region of use. This could indeed be another matter of study.

### Conclusions

If privacy issues in diabetes mobile apps are not dealt with carefully, users may unwillingly and unknowingly share very sensitive private data. Therefore, it is crucial that all stakeholders are involved in the development of diabetes apps from the very beginning of the process in order to ensure apps’ absolute compliance with data protection regulations and user privacy.

As the economic value of personal data increases [59], a completely new business model for apps has emerged: users pay for the usage of an app with their data, which is then sold to third parties, such as advertising clients [60]. The lesson to be learned is that there is a price to pay in exchange for free apps, usually at the expense of privacy. Consequently, new control measures are needed to enable users to decide which personal information they are willing to disclose in return for a certain service [61].

The importance of personal data protection laws and their endorsement are of utmost importance. Well-designed privacy policies may protect individuals by requiring consent for the collection, use, disclosure, or retention of sensitive personal and health information, and they may regulate the use of these extremely sensitive data, allowing users to modify their information as well as to revoke their previous consent.

Therefore, we recommend proper training for users, enforcement of strict data protection laws by governments and regulatory bodies, much tougher security policies and protocols in both Android apps and the Google Play Store, and the implication and supervision of all stakeholders in the app development process.

## Appendix

Multimedia Appendix 1 Qualitative results of case studies.

Multimedia Appendix 2 Top 10 Android’s dangerous permissions identified.

Multimedia Appendix 3 Comma-separated values files.

## Abbreviations

API: application programming interface
CSV: comma-separated values
DM: diabetes mellitus
mHealth: mobile health
