# Supplementary material for: Analysis of Diabetes Apps to Assess Privacy-Related Permissions: Systematic Search of Apps
Source: JMIR Diabetes. 2021 Jan 13;6(1):e16146. doi: 10.2196/16146 (PMC7840294; doi:10.2196/16146)
Supplement: Multimedia Appendix 1 [file diabetes_v6i1e16146_app1.docx]

## **Multimedia Appendix III. 2-case studies qualitative results**

The principal difference between the two selected apps resided in that *Diabetes:M* contains advertising and is based on a *freemium* model (i.e., the app’s basic capabilities are free but it contains advertising, while in the app’s paid version the advertising disappears and the app functionalities are expanded). On the other hand, *BeatO Smart Diabetes Management* fully-featured version is completely free of cost and claims not to contain advertising. The idea behind was, in addition to complete the previous results, explore whether there was a correlation between advertising and a more aggressive usage of dangerous permissions.

This is a Multimedia Appendix to a full manuscript published in the J Med Internet Res. For full copyright and citation information see http://dx.doi.org/10.2196/jmir.16146

#### ***First case-study: Diabetes:M (the app declares that contains advertising)***

*Diabetes:M* was installed in a mobile phone with Android 9.0 “Pie” version. The reason for choosing this app was that it was very representative: it had 500,000+ downloads, counted with 18,000+ reviews and possessed an excellent average rating of 4.6. In brief, it was a very popular and well-rated app that contained advertising.

Table 5 below shows *Diabetes:M*’s short description, the app’s dangerous permissions automatically retrieved by the web scraper from the API of 42Matters, as well as *Diabetes:M*’s dangerous permissions directly and manually retrieved from the Google Play Store.

| *Example of a freemium diabetes app with declared advertising:  Diabetes:M* | | |
| --- | --- | --- |
| ***App’s description (app’s declared purpose)*** | ***Android dangerous permissions***  ***(automatically retrieved from 42Matters)*** | ***Requested permissions (manually retrieved from the Google Play Store)*** |
| *Designed for both smartphones and tablets, this application will help you manage your diabetes better and keep it under control. Whether you are Type 1 or Type 2, have Gestational Diabetes or just want to help and monitor a family member, this is the logbook app for you.*  *The application tracks almost all aspects of the diabetes treatment and provides you and with detailed reports, charts and statistics. You can send the reports to your supervising physician via email.*  *Diabetes:M also gives you various tools, so you can find the trends in blood glucose levels and allows you to calculate normal and prolonged insulin boluses using its highly effective, top-notch bolus calculator.*  *It also has a vast nutrition database, to help you keep track of your food intake and nutrition information, as well as exercise time. Never forget another check with our simple but powerful reminders system.*  *Diabetes:M can analyze the values from the imported data from various glucometers and insulin pumps via the exported files from their respective diabetes management software systems.* | ***Access Coarse Location*** | ***Storage/Photos/multimedia/files:***   - *Modify or delete the contents of the USB storage* - *Read the contents of the USB storage*   ***Contacts/Identity***   - *Find accounts on the device*   ***Location***   - *Precise location (GPS and network-based)* - *Approximate location (network-based)*   ***Camera***   - *Take pictures and videos*   ***Others***   - *Receive data from Internet* - *Create accounts and set passwords* - *Connect and disconnect from Wi-Fi* - *Allow Wi-Fi Multicast reception* - *View Wi-Fi connections* - *Full network access* - *Control vibration* - *Access Bluetooth settings* - *Control Near Field Communication* - *Run at startup* - *Prevent device from sleeping* - *View network connections* - *Pair with Bluetooth devices* - *Use accounts on the device* |
|  | ***Access Fine Location*** |  |
|  | ***Write External Storage*** |  |
|  | ***Get Accounts*** |  |
|  |  |  |
|  | ***Camera*** |  |

Table 5. Diabetes:M’s description and requested dangerous permissions

Table 5 demonstrates that there is a perfect match between the app’s dangerous permissions automatically retrieved by the web scraper and the permissions manually obtained from the Google Play Store, consequently validating both that the web scraper works properly and that 42Matters is a reliable data source. According to *Diabetes:M*’s declared purpose, it would be reasonable for the app to require the **Get Accounts** permission (for instance, to send information to the doctor), as well as the **Write External Storage** dangerous permission (to store clinical data related to the app). However, it is difficult to find a solid explanation that justifies why would the app, for its declared purpose, need access to the **Access Fine Location** and **Access Coarse Location** dangerous permissions.

| 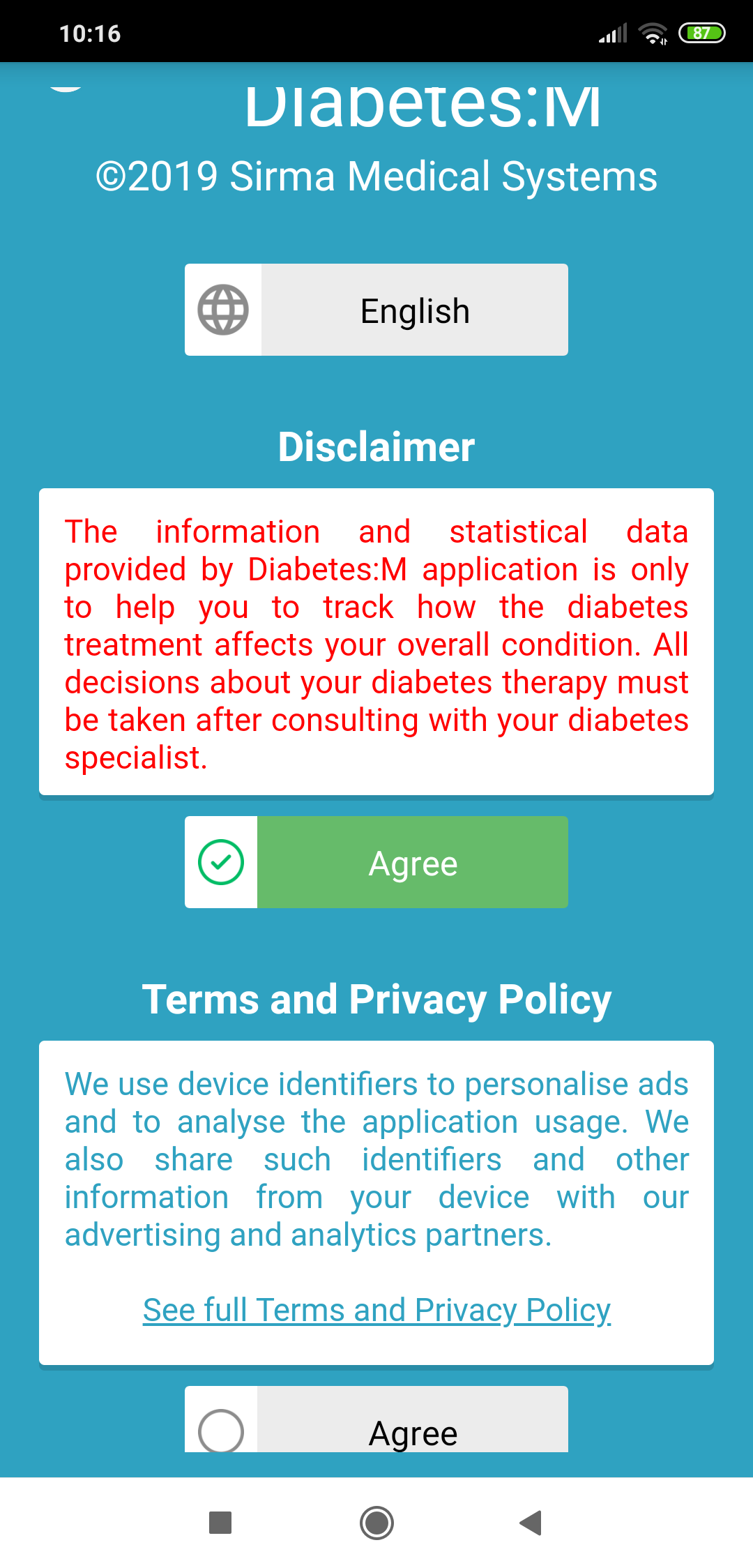 | 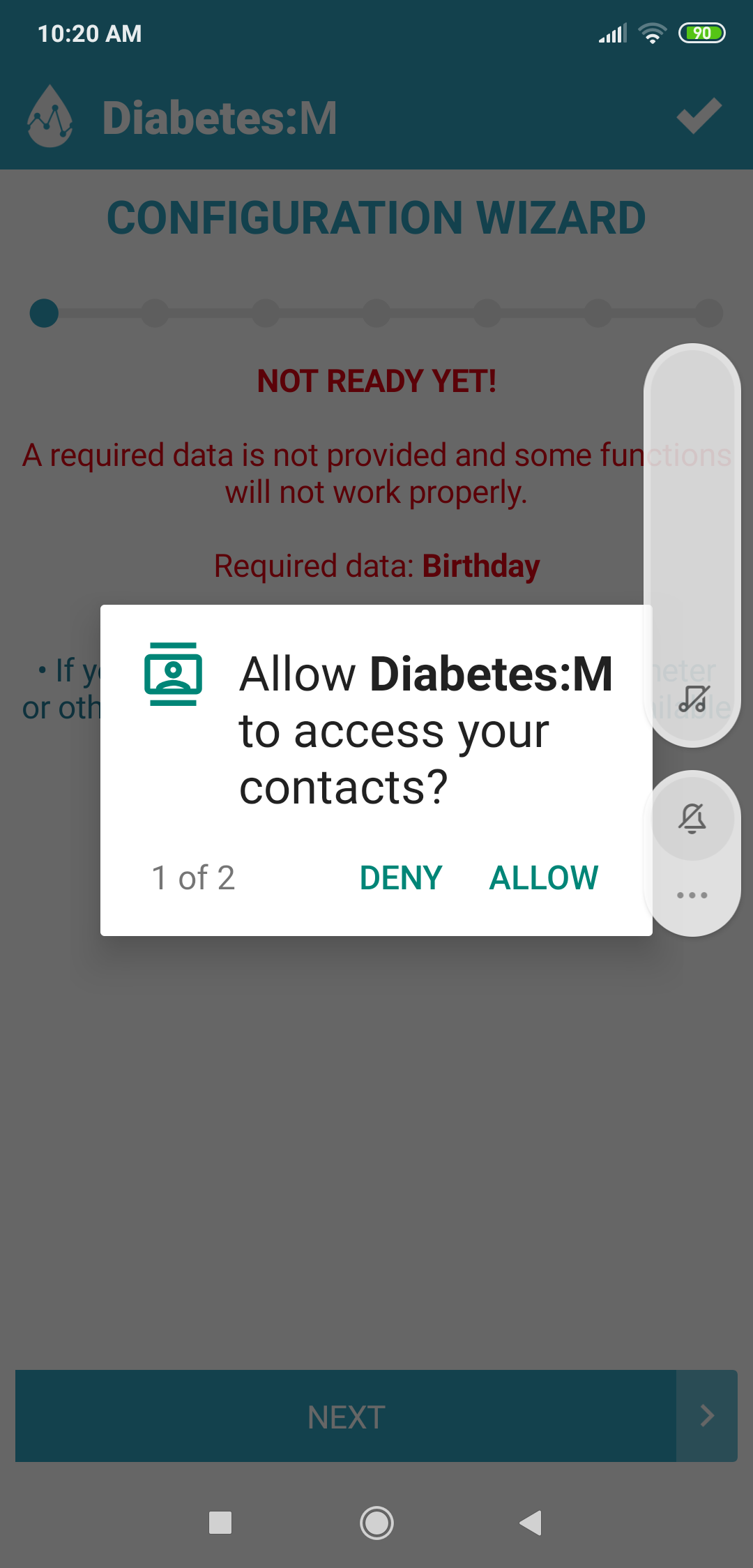 | 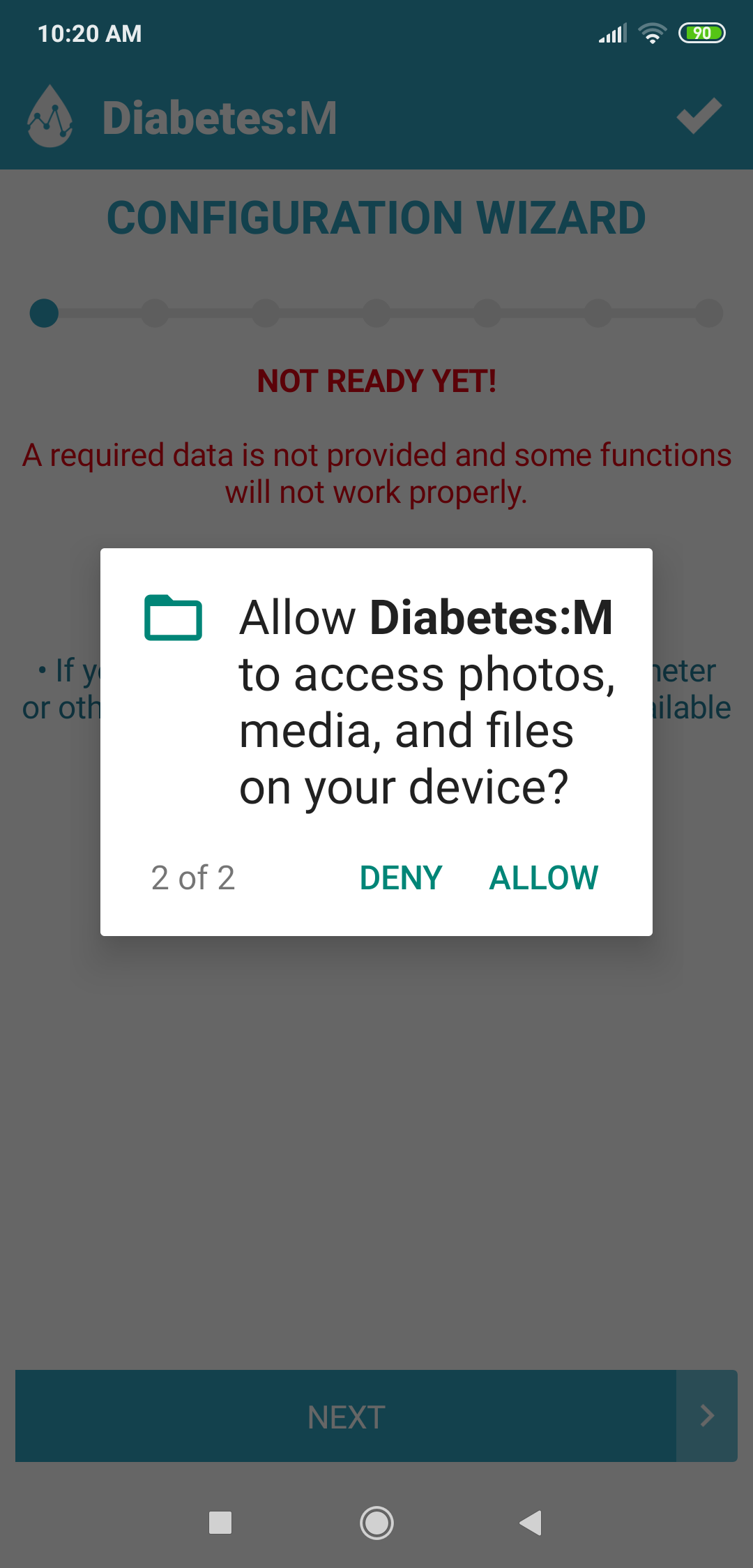 |
| --- | --- | --- |
| 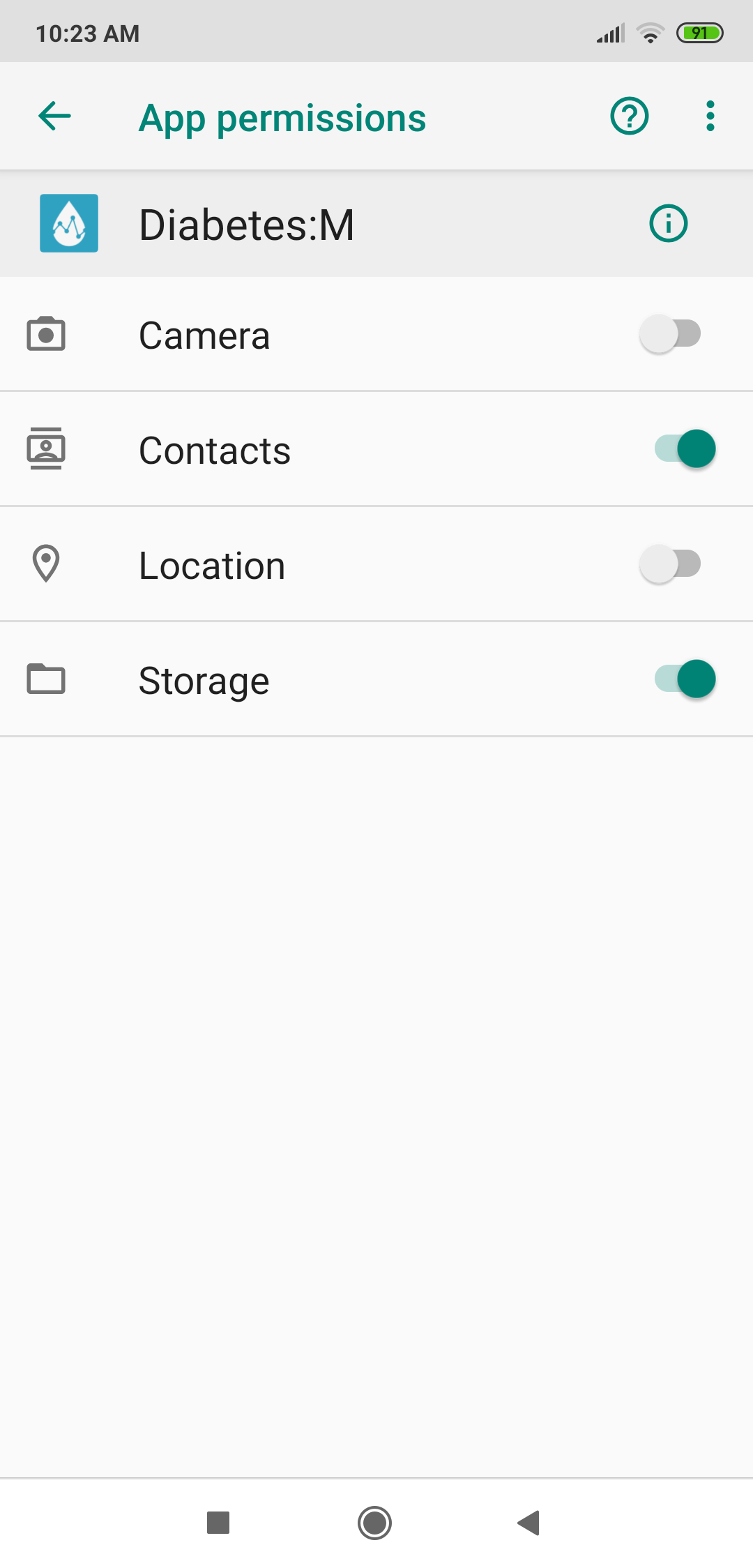 | 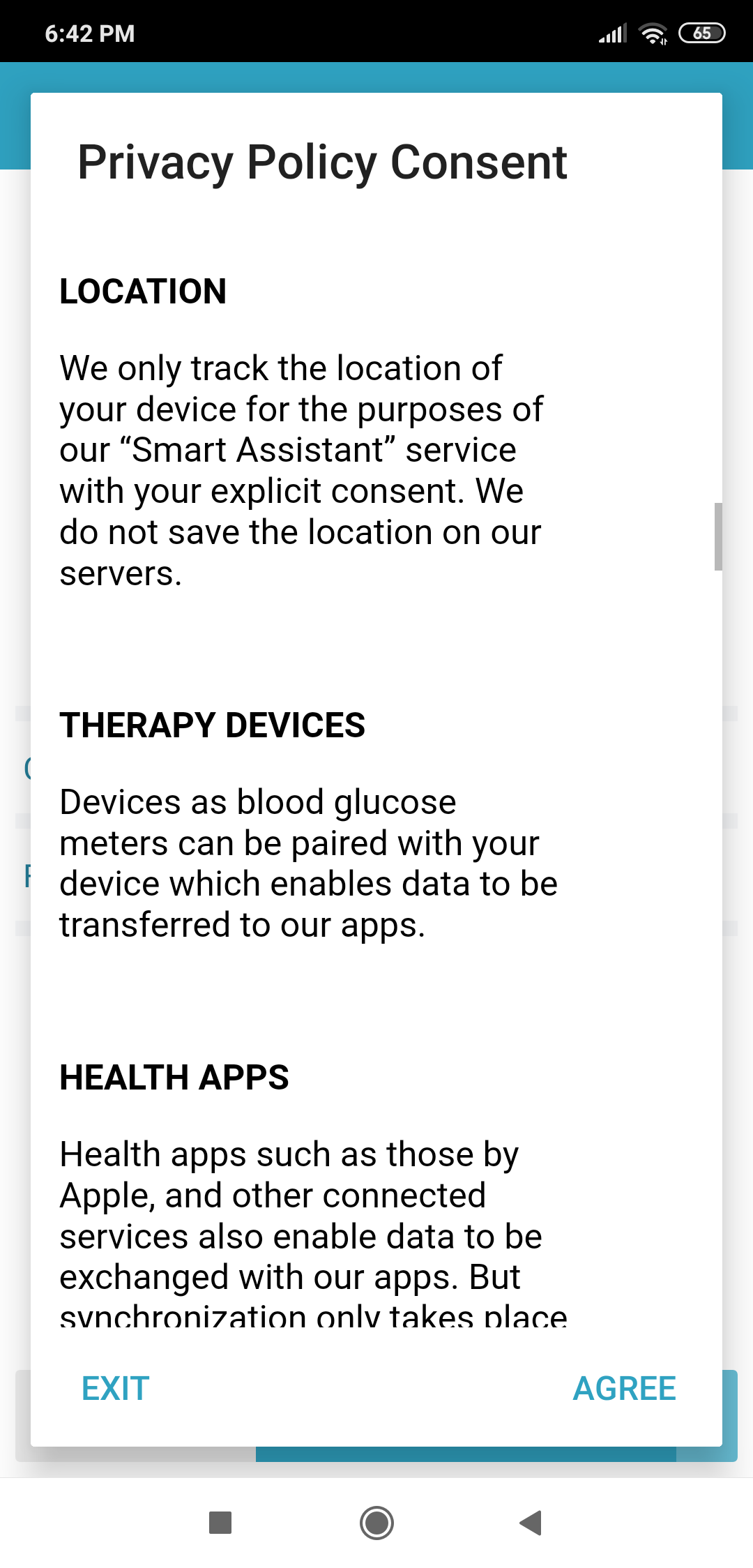 | 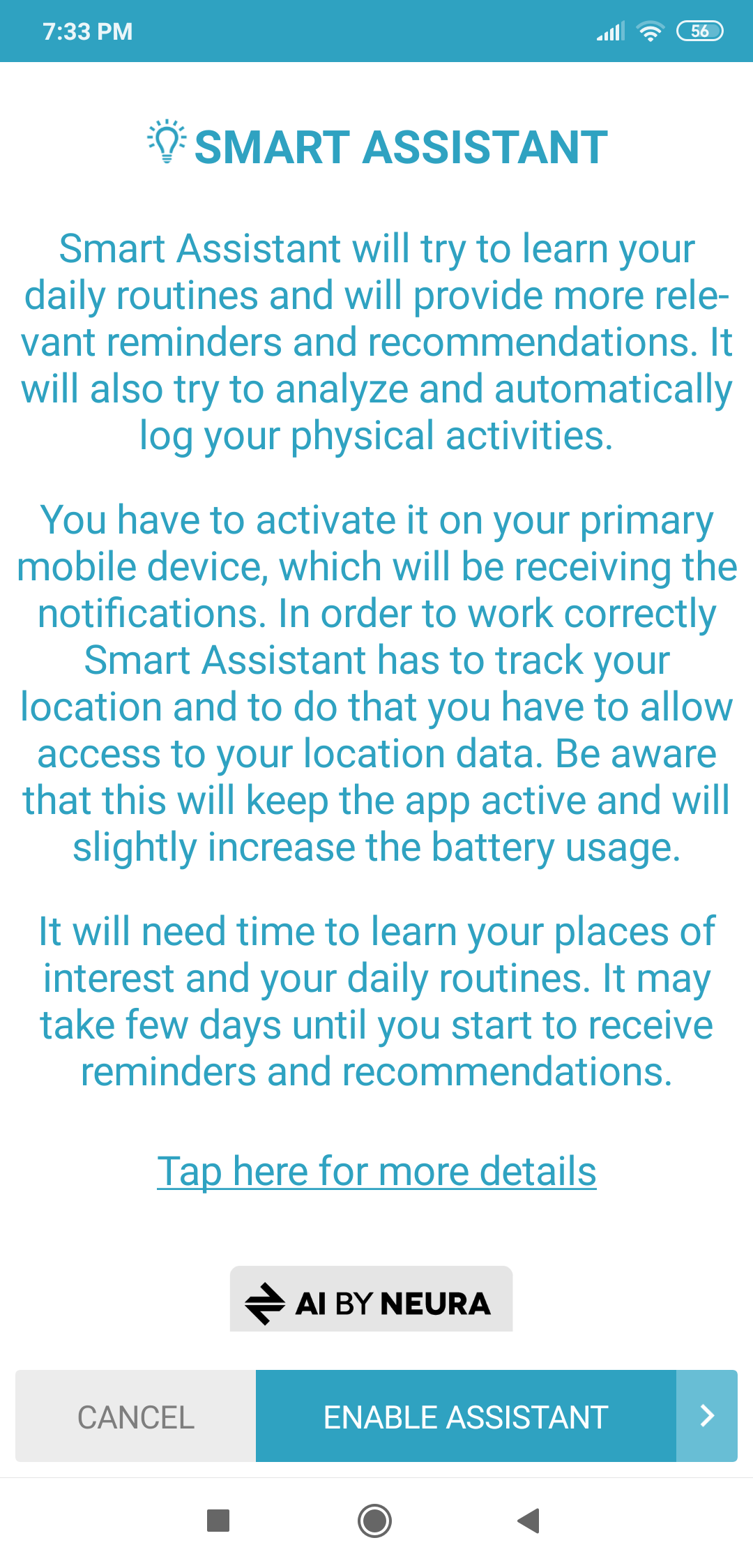 |
| 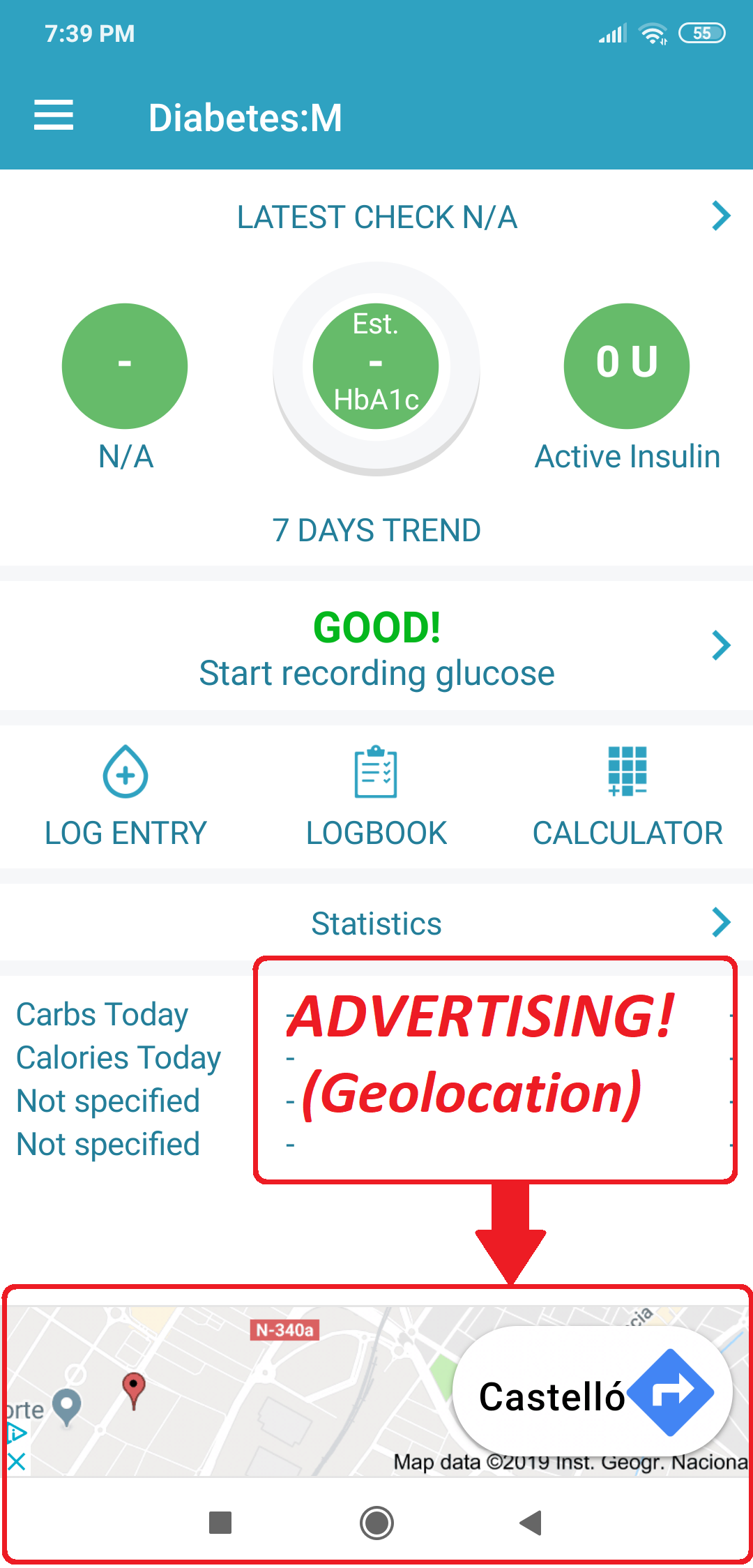 | 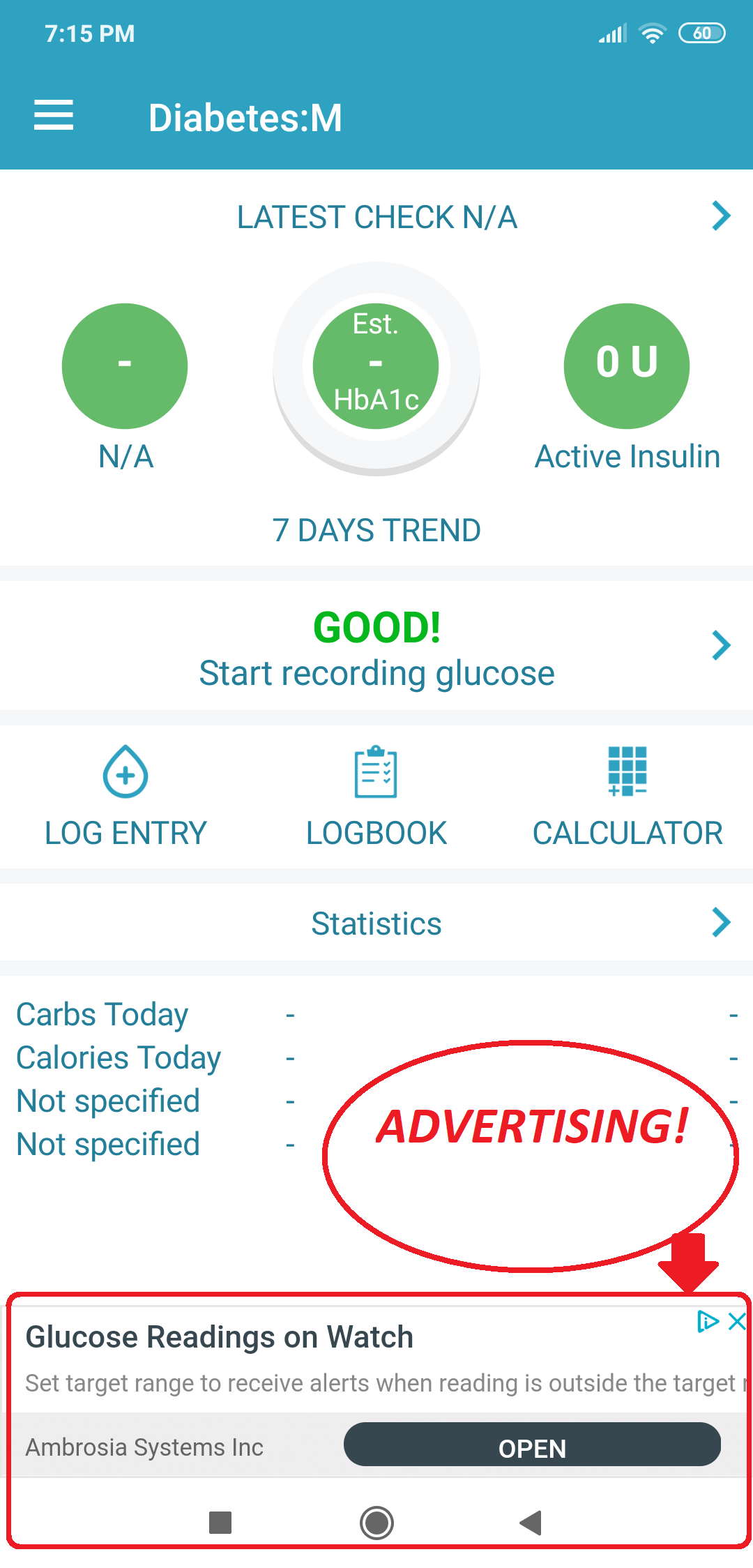 | 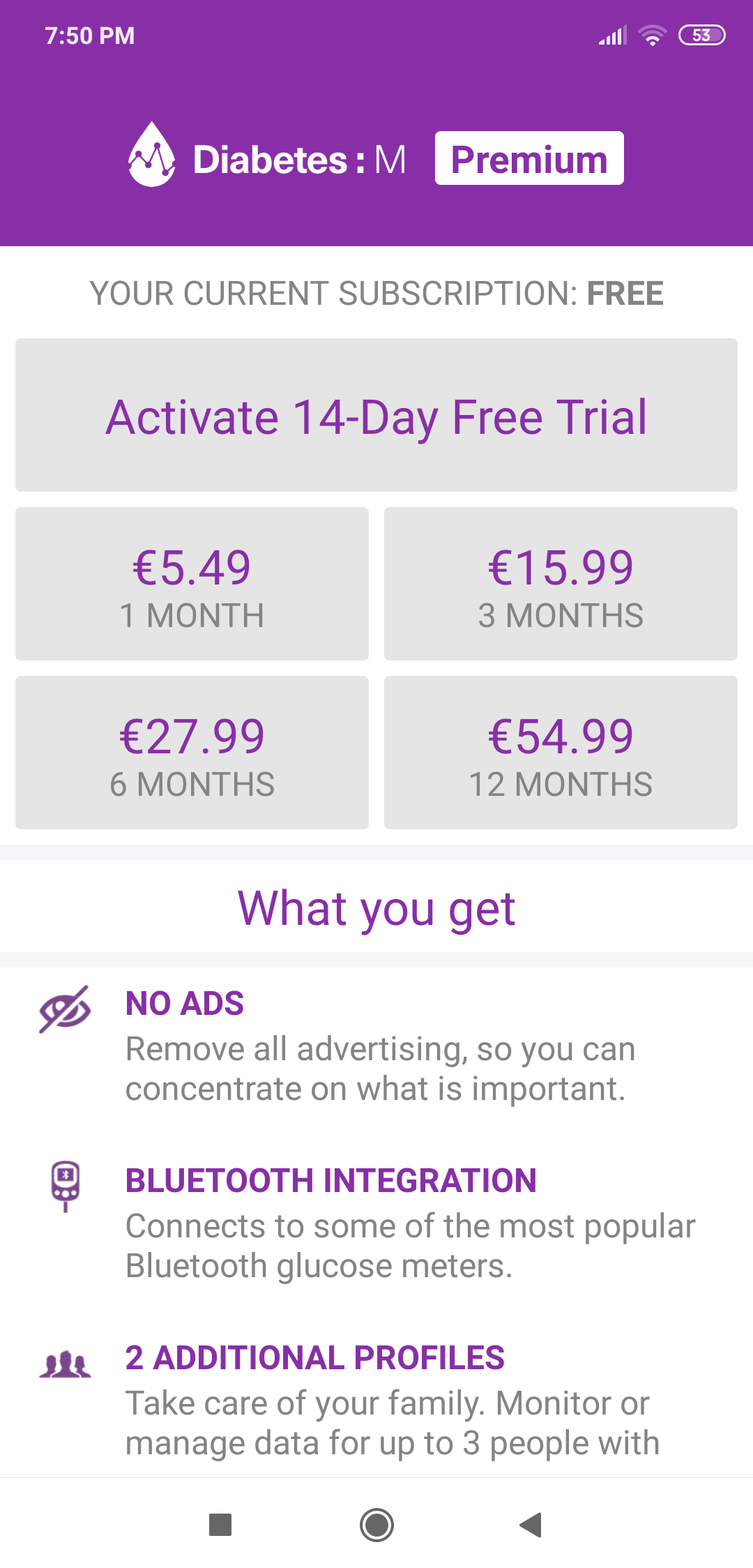 |

Table 6. Screenshots of Diabetes:M showing the app’s privacy policy, required dangerous permissions, continuous popping-up advertising, and the Premium subscription option

The most likely explanation is that the developer’s business model is based on marketing the app for free at the expense of generating revenues by including customized advertising in the app. In this sense, it should be noted that more than 1 million apps use Google AdMob, a smart monetization platform for Android that helps app developers to maximize revenue from ads. In particular, according to Google:

***“AdMob shows ads from millions of Google advertisers in real time. App developers can monetize users quickly with AdMob, by showing ads to them in more than 200 markets. Well-placed, well-targeted ads in apps, particularly free apps, can achieve good click-through rates. For the developer it is easy to add the code to deliver ads, and Google AdMob takes care of the rest: finding and delivering relevant ads to your app from any of Google’s advertiser demand across Google AdMob, Google Ads, and the authorized buyers.***” [60]

This is absolutely in line with our research findings, that demonstrate that 95.4% of the apps included in our study are free and 40% of them openly claim to contain advertising. What Google or the app developers do not explicitly mention is that, unless the user disables the permissions in many different and complex settings, not only present in the app but in the entire Android operating system, the users’ private data will be used to commercialize the aforementioned customized advertising. The immediate consequence is that app developers, by using advertising, contribute either directly or indirectly to disclose very critical personal data to unknown third-parties.

Although the possibility of sharing critical data with third-parties is mentioned once during *Diabetes:M*’s installation process, for instance it is not expressly mentioned in the app developer’s privacy policy website (https://sites.google.com/view/diabetes-m-userguide/terms-and-privacy).

Regarding the use of the **Camera** dangerous permission, the only reasonable need would be for taking pictures or scanning the barcodes of diabetes medicines, for food logging, or for sharing body-parts pictures with the doctor to obtain a diagnostic. However, no mention to these potential use-cases could be found in the app’s description.

#### ***Second case-study: BeatO Smart Diabetes Management (the app declares it does not contain advertising, although it actually does)***

In addition, the *BeatO Smart Diabetes Management* app was installed in the same mobile phone. The reason for choosing this app was very similar to the previous one. It was very representative, since it had 100,000+ downloads, counted with 4,700+ reviews and possessed a positive average rating of 4.5. Although the app’s description states that it does not contain advertising, it actually does.

| *Example of a free diabetes app declaring no advertising (but actually containing it):* *BeatO Smart Diabetes Management* | | | |
| --- | --- | --- | --- |
| ***App’s description (summarized)*** | ***Android dangerous permissions***  ***(automatically retrieved from 42Matters)*** | ***Requested permissions (manually retrieved from the Google Play Store)*** |  |
| *BeatO is India’s leading healthcare App to help you monitor and manage diabetes the smart way. It is the only diabetes app you need to check your sugar levels easily on the go for better health.*  *Trusted by over 1 Lac diabetic users, here’s why BeatO is the most popular Diabetes Management app:*  ***BeatO SMART Blood Sugar tracker:*** *Use BeatO’s affordable and unique one touch glucometer (ISO and CE certified) with BeatO App for accurate and easy blood sugar level readings. It is extremely small in size and simply plugs into the audio port of your smartphone for instant accurate results. Alternatively, you can also manually log blood glucose readings from other Glucometers in BeatO App logbook.*  ***FREE Doctor and Dietician Consultations:*** *FREE BeatO care round the clock! Consult top doctors and our team of medical experts (dieticians) who help you to constantly monitor your sugar levels and can be reached out 24/7 on call or chat. No more waiting for appointments.*  ***Record, Analyze and Share your Blood Glucose readings:*** *BeatO App uses color coding to depict high, low and normal blood sugar levels. All blood glucose test results are compiled in the app logbook, can be analyzed with simple graphs and shared with your family, doctor and trusted contacts through automatic SMS alerts.*  ***Sync your fitness tracker:*** *Find out the calories you burn by syncing your fitness tracker to track your steps. BeatO fitness tracker is compatible with Google Fit to show a unified view of your health and activity data.* | ***Access Coarse Location*** | ***Storage/photos/multimedia/ files:***   - *Modify or delete the contents of the USB storage* - *Read the contents of the USB storage*   ***Contacts/Identity***   - *Find and read accounts and contacts on the device*   ***Microphone***   - *Record audio*   ***Phone***   - *Read phone status and identity*   ***Location***   - *Precise location (GPS and network-based)* - *Approximate location (network-based)*   ***Camera***   - *Take pictures and videos*   ***Others***   - *Receive data from Internet* - *Allow Wi-Fi Multicast reception* - *Run at startup* - *View network connections* - *Full network access* - *Change your audio settings* - *Control vibration* - *Access Bluetooth settings* - *Draw over other apps* - *Prevent device from sleeping* - *Connect and disconnect from Wi-Fi* - *Pair with Bluetooth devices* |  |
|  | ***Access Fine Location*** |  |  |
|  | ***Write External Storage*** |  |  |
|  | ***Read External Storage*** |  |  |
|  | ***Get Accounts*** |  |  |
|  | ***Camera*** |  |  |
|  | ***Read Contacts*** |  |  |
|  | ***Read Phone State*** |  |  |
|  | ***Record Audio*** |  |  |

Table 7. BeatO Smart Diabetes Management’s description and requested dangerous permissions

As in the previous case-study, Table 7 above shows the app’s description, and what dangerous permissions the app requests according to both 42Matters and to the Google Play Store. Once again, there is a perfect match between the information independently retrieved from the two platforms.

Subsequently, from Table 8 below, it is possible to conclude different issues regarding the app *BeatO Smart Diabetes Management.*

| 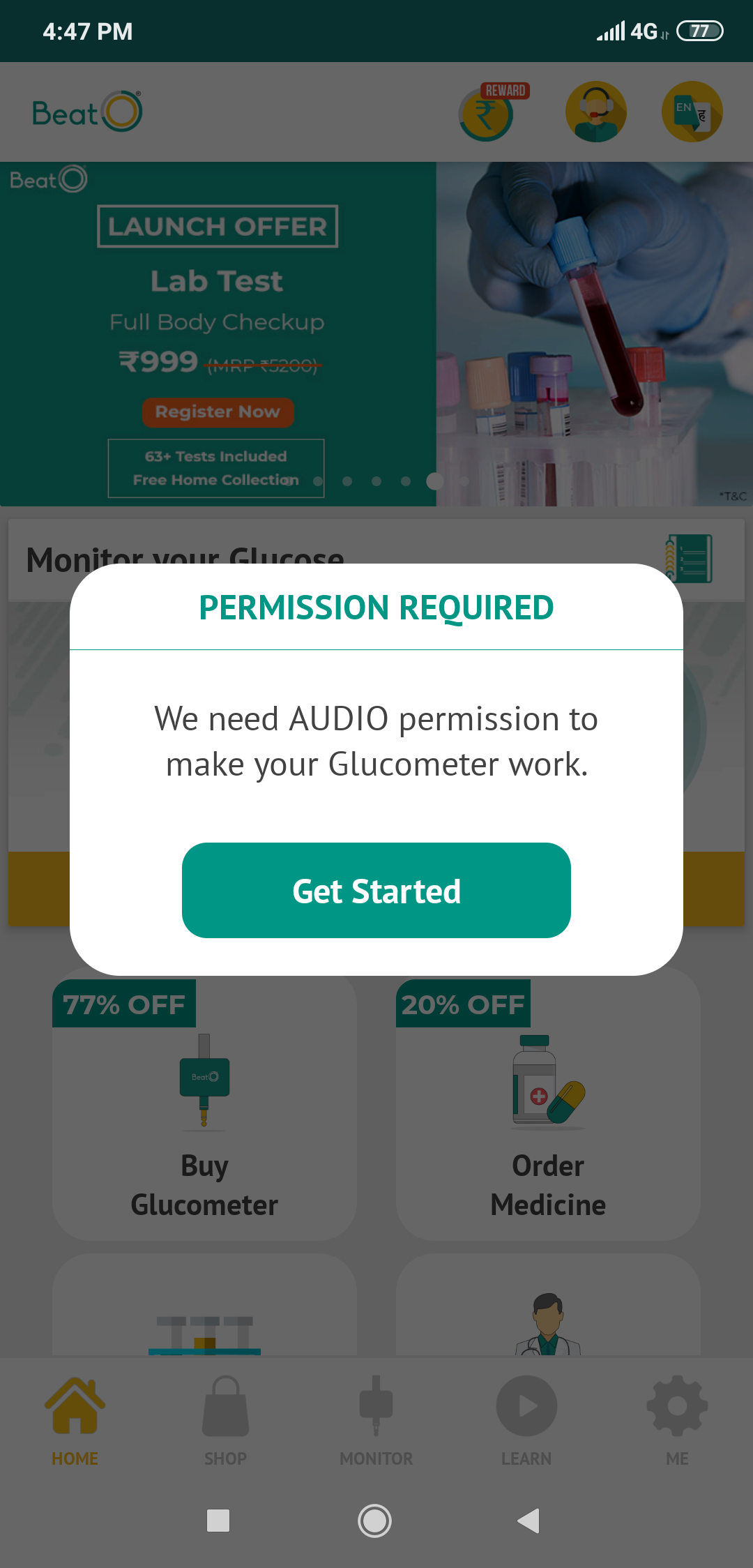 | 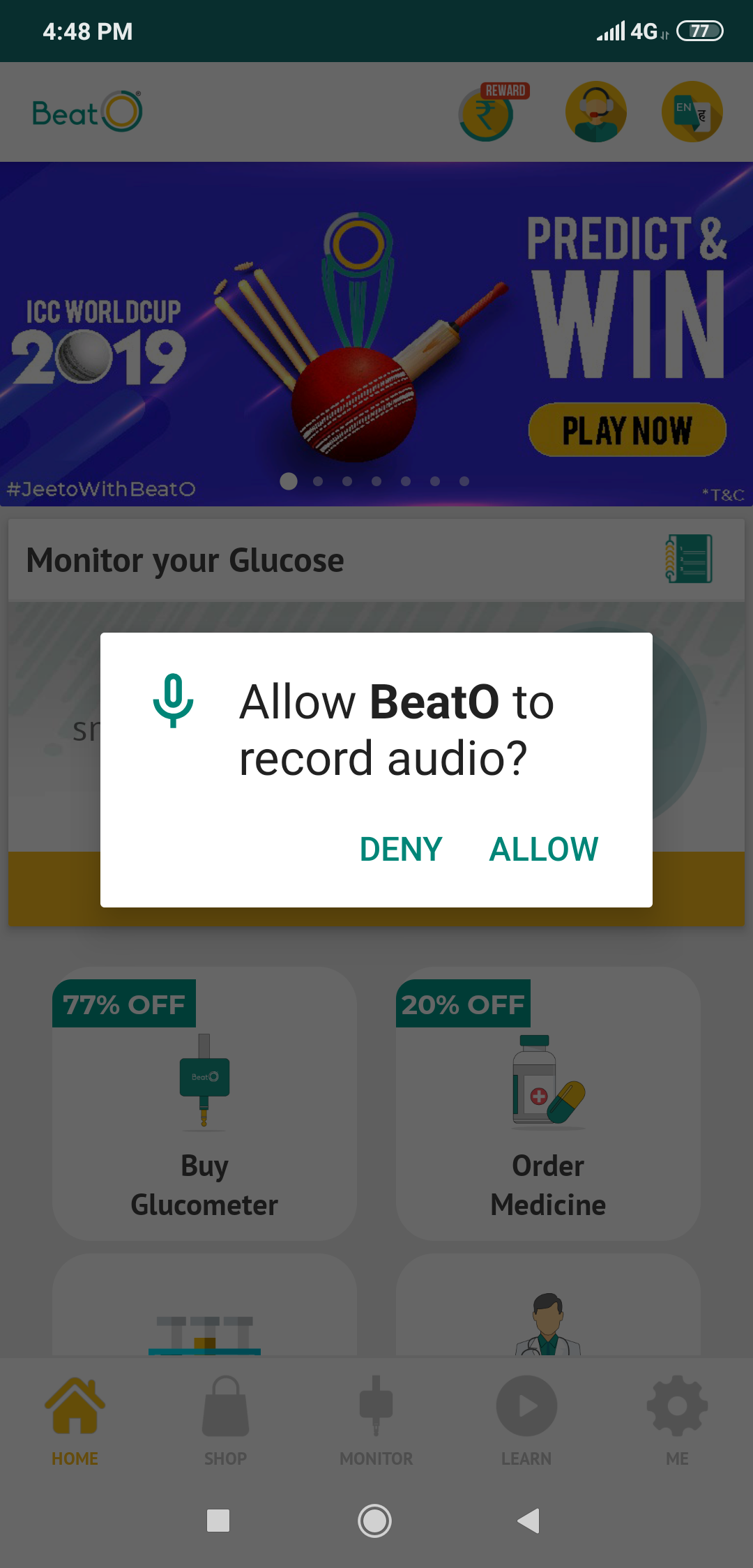 | 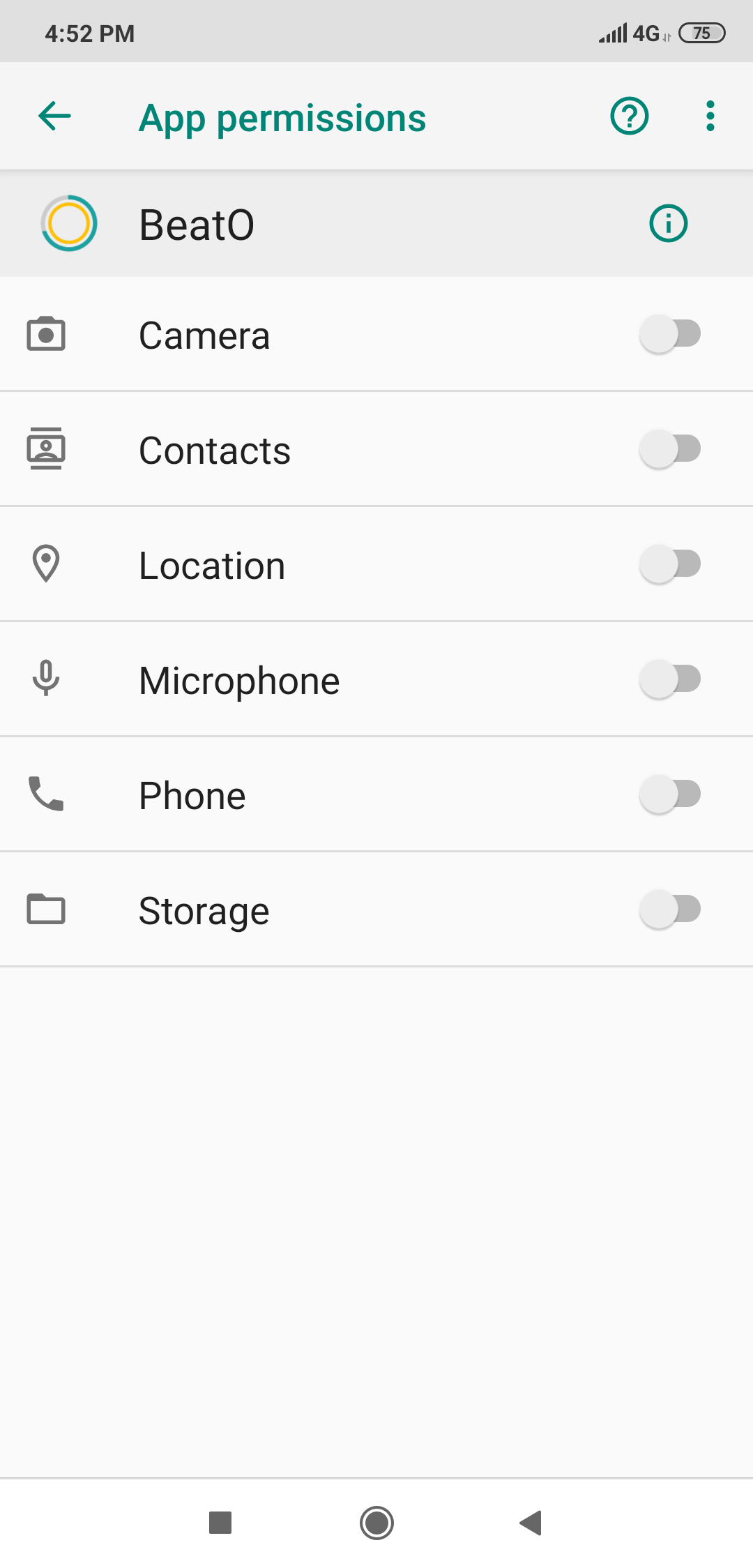 |
| --- | --- | --- |
| 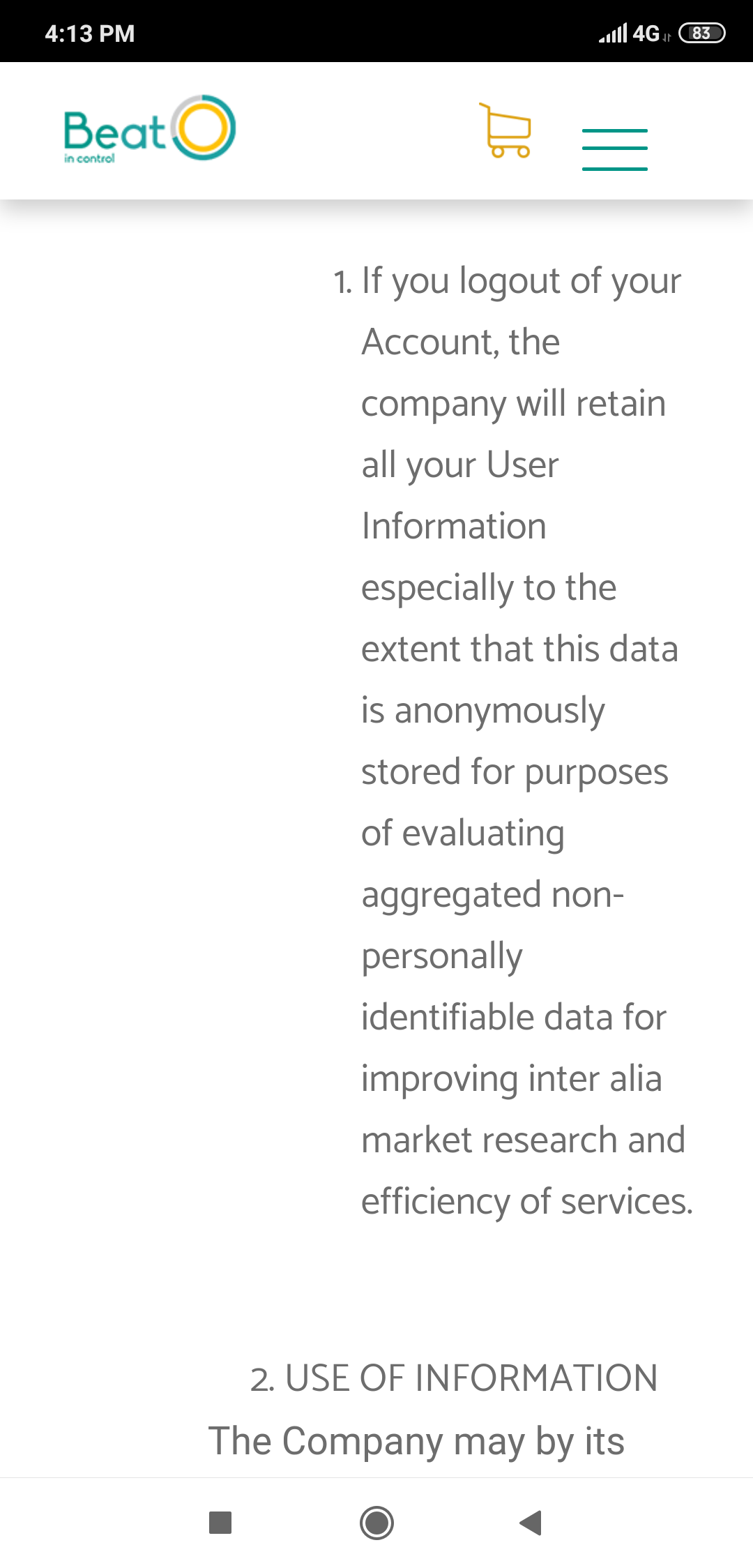 | 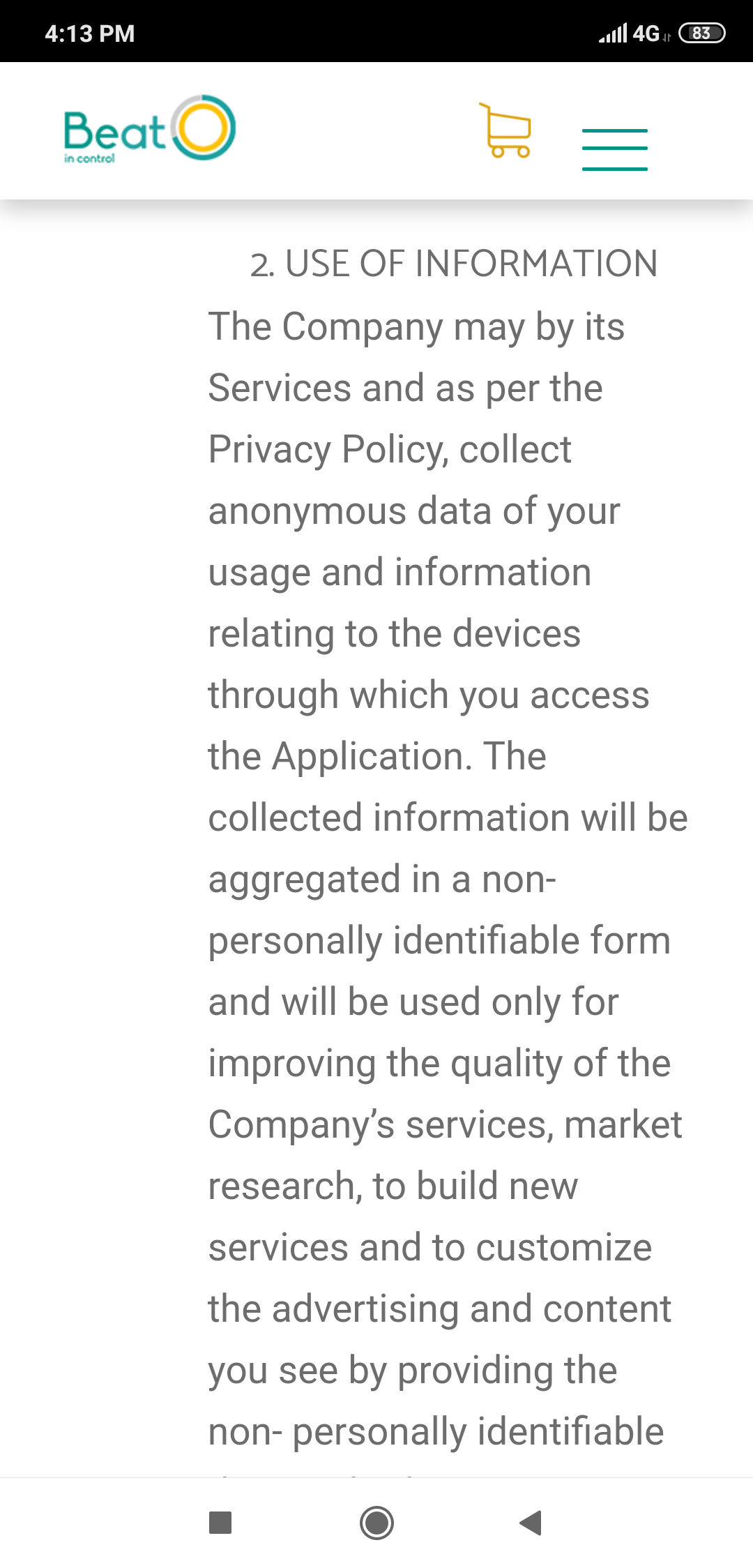 | 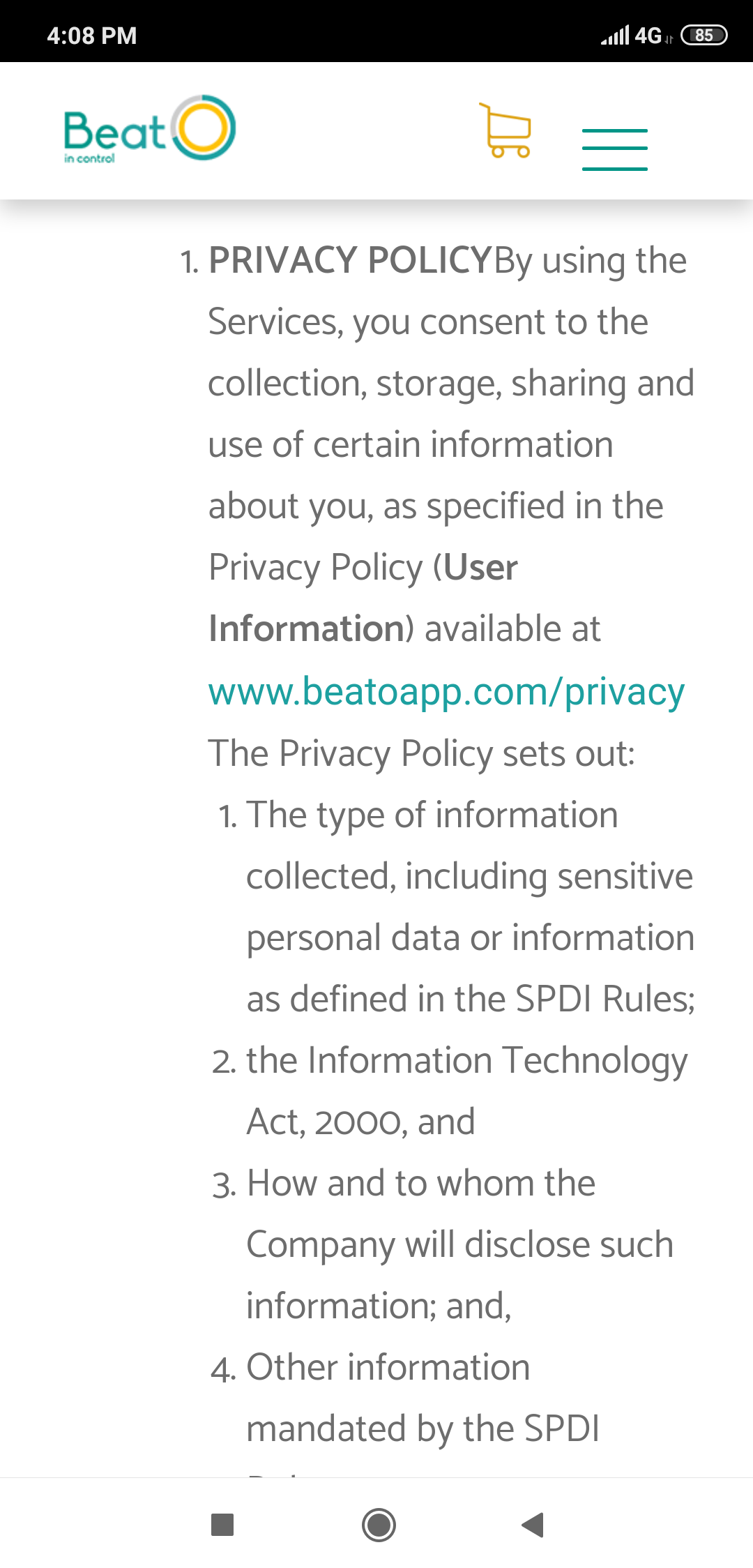 |
| 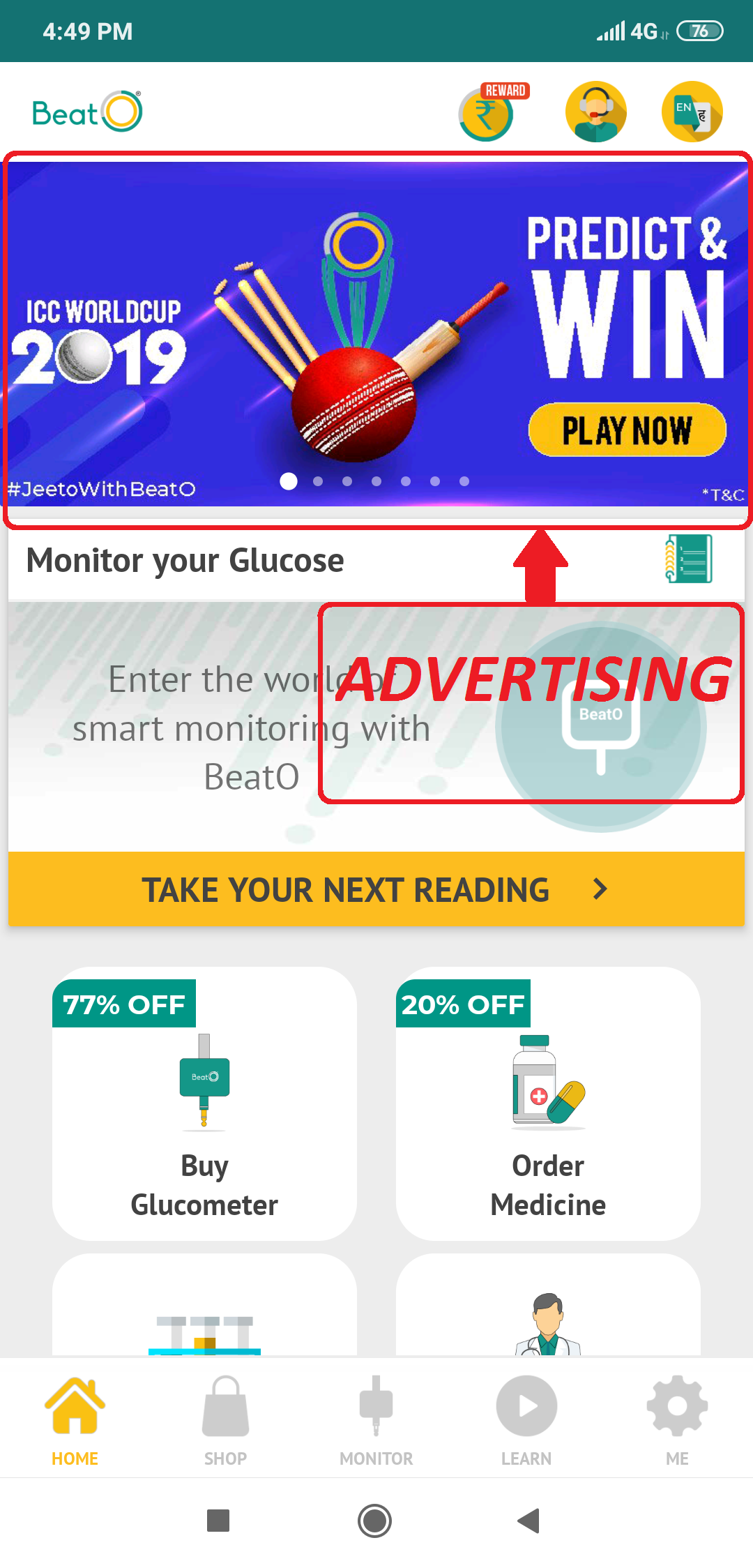 | 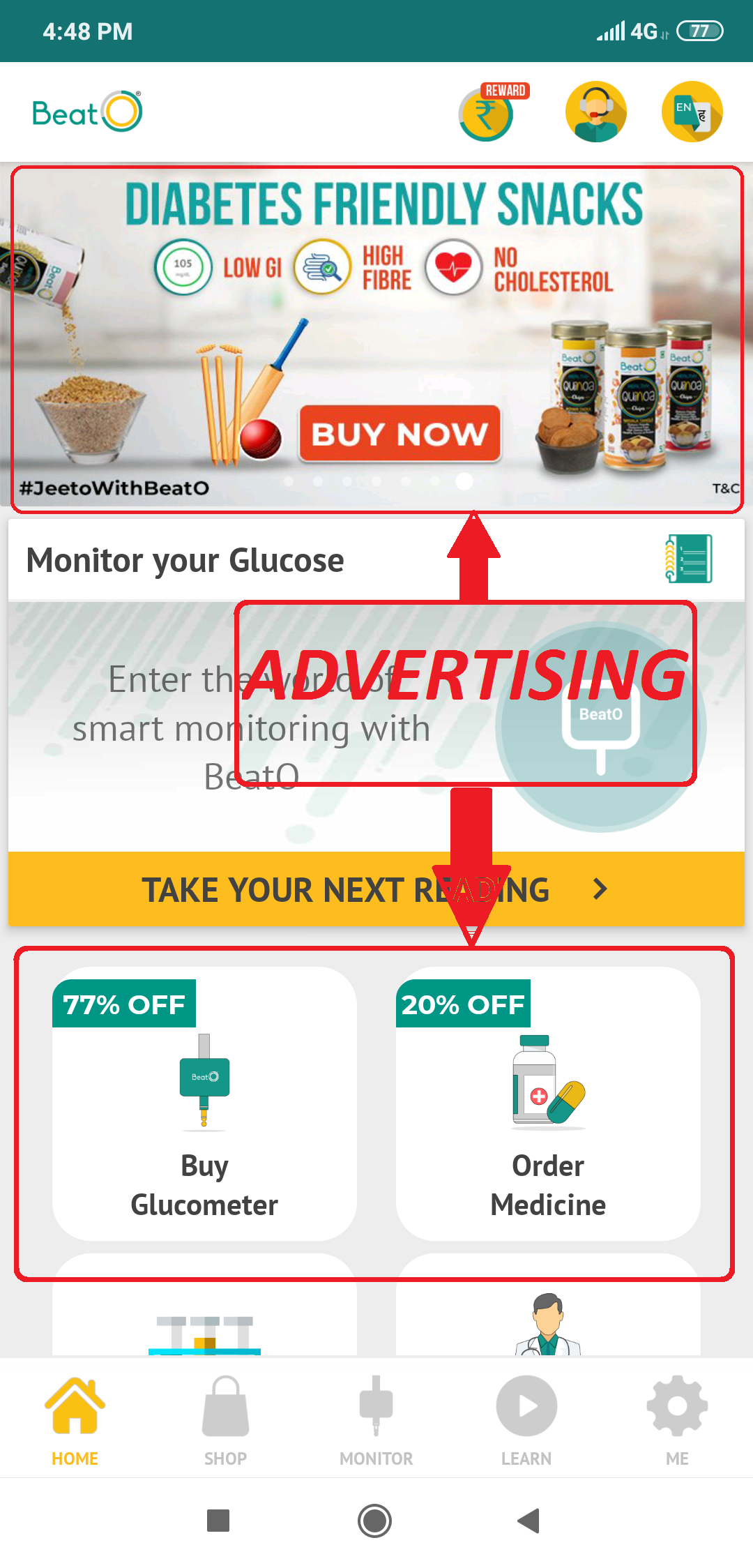 | 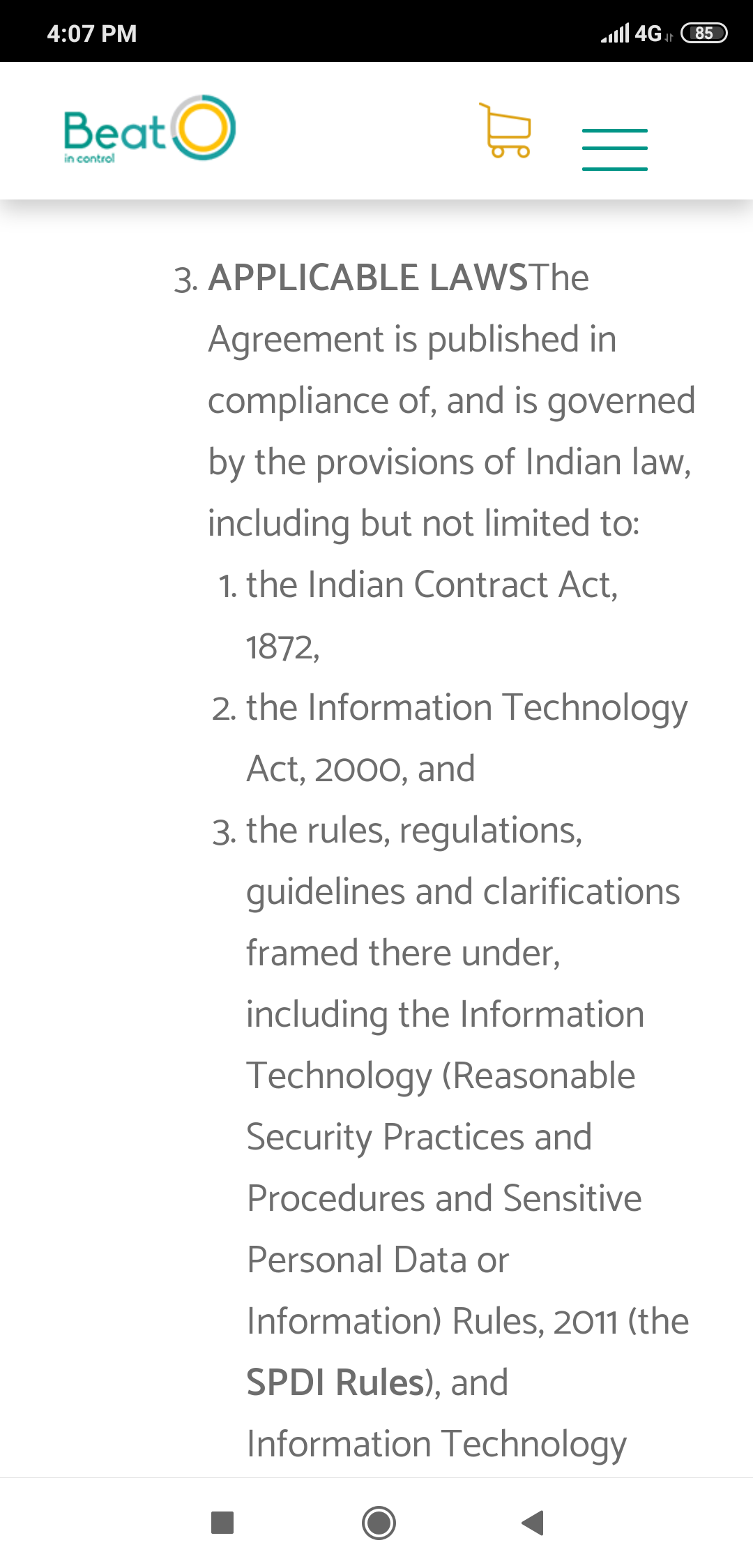 |

Table 8. Screenshots of BeatO Smart Diabetes Management showing the app’s privacy policy, required dangerous permissions and the advertising

First, in an attempt to justify *BeatO Smart Diabetes Management*’s need for dangerous permissions, the relationship between the app’s requested permissions and the app’s description is discussed. The **Audio** permission is most likely needed to allow the connection with the glucometer. While it is true that some glucometers still use the mobile phone’s audio jack as a physical connector, it is also certain that, once the audio permission has been granted, users have no longer any control over whether the app is truly pairing the mobile with the glucometer, or, on the contrary, recording the users’ voice and phone calls without their explicit knowledge and consent. Regarding the **Access Coarse Location** and **Access Fine Location** permissions, there might be an actual need to request them, but only if the user utilizes the app’s fitness tracker functionality and Google Fit. Moreover, it would be logical to demand the **Write External Storage** and **Read External Storage** permissions to retrieve and store actual diabetes-related information, such as the glucometer readings, consumed food calories and logs. In addition, although not strictly necessary, the **Read Contacts** permission might be requested to facilitate and accelerate contacting the team of medical experts. Moreover, as already mentioned in the *Diabetes:M* case-study, the **Camera** permission could be useful for food logging, although this purpose is not even listed in BeatO Smart Diabetes Management’s description.

However, a disclaimer should be made: any potential justifications for requesting the aforementioned permissions do not necessarily exclude the possibility of the app developer making a fraudulent or non-transparent use of the users’ critical data. To support this claim, it must be remarked that we could not find any reasonable or legitimate need for requesting the **Read Phone State** dangerous permission, which for instance allows accessing the device’s IMEI number (a unique number that univocally identifies a mobile phone) or knowing the existence of an ongoing phone call. In addition, the app apparently inappropriately may request the **Get Accounts** permission, which allows retrieving the user’s device accounts in services such as Google, Facebook or Instagram, to name a few. In brief, it is very difficult to verify whether there is an actual need for requesting dangerous permissions.

Second, several screenshots illustrating the app’s terms of use are shown, in which it is explicitly mentioned that the app may share the users’ data (anonymized, according to the app, although this extreme could not be verified) for advertising, marketing or research purposes, or even with undetermined third-parties.

Next are presented some screenshots related to the app’s privacy policy, which complements what the app already stated in the terms of use. Subsequently, the shown third-party policy screenshots keep elaborating on the same issues: critical personal data may be exploited or shared for different purposes. Fourth, screenshots displaying continuous advertising in the app are presented: consequently, it is quite obvious that the app actually contains advertising, contradicting its description.

Finally, the last screenshot shows the alleged app’s compliance with the Indian Government’s Data Laws, but at this point it should be remarked that the app was installed in Spain, a country which data protection laws fully adhere to and enforce the ones of the European Union. Therefore, it remains unclear whether *BeatO Smart Diabetes Management* is fully compliant with the European Union’s stricter (from the users’ data protection perspective) General Data Regulation Protection [61].
