# Supplementary material for: Analysis of Diabetes Apps to Assess Privacy-Related Permissions: Systematic Search of Apps
Source: JMIR Diabetes. 2021 Jan 13;6(1):e16146. doi: 10.2196/16146 (PMC7840294; doi:10.2196/16146)
Supplement: Multimedia Appendix 2 [file diabetes_v6i1e16146_app2.docx]

## **Multimedia Appendix I. Top 10 Android’s dangerous permissions identified.**

This is a Multimedia Appendix to a full manuscript published in the J Med Internet Res. For full copyright and citation information see http://dx.doi.org/10.2196/jmir.6146

| Description of this research’s top 10 dangerous permissions | |
| --- | --- |
| ***Permission*** | ***Description*** |
| ***Write External Storage*** | Allows apps to write to the mobile’s external storage (i.e., an external memory card) |
| ***Read External Storage*** | Allows apps to read from the device’s external storage |
| ***Access Coarse Location*** | Allows apps to access the approximate location (using cellular base stations and Wi-Fi hotspots) |
| ***Access Fine Location*** | Allows apps to access the device’s exact location (using GPS) |
| ***Camera*** | Allows apps to use the phone’s camera to take photos and record videos |
| ***Get Accounts*** | Allows access to the complete list of the device’s accounts |
| ***Read Phone State*** | Allows read-only access to the phone state, including the mobile phone number, the current cellular network information, and the status of any ongoing calls |
| ***Record Audio*** | Allows apps to use the mobile’s microphone to record audio |
| ***Call Phone*** | Allows an application to initiate a phone call without the user’s confirmation |
| ***Read Contacts*** | Allows an application to read the user’s contacts data |

Description of this research’s top 10 required Android dangerous permissions
