# Supplementary material for: Analysis of Diabetes Apps to Assess Privacy-Related Permissions: Systematic Search of Apps
Source: JMIR Diabetes. 2021 Jan 13;6(1):e16146. doi: 10.2196/16146 (PMC7840294; doi:10.2196/16146)
Supplement: Multimedia Appendix 3 [file diabetes_v6i1e16146_app3.docx]

## **Multimedia Appendix II. CSV files**

We include the two most important data-files of this research: the CSV file containing the 497 apps and related metadata; and the Boolean matrix of dangerous permissions.

This is a Multimedia Appendix to a full manuscript published in the J Med Internet Res. For full copyright and citation information see http://dx.doi.org/10.2196/jmir.16146
